# Policies and programmes to improve preconception nutrition in South Asia

**DOI:** 10.1016/j.lansea.2025.100589

**Published:** 2025-05-01

**Authors:** Avishek Hazra, Tashi Choedon, Monica Shrivastav, Raj Kumar Verma, Cheshta Gulati, Dhammica Rowel, Abner Daniel, Preetu Mishra, Naveen Paudyal, Naureen Arshad, Muhammad Salman, Wisal Khan, Khadija Khalif Osman Warfa, Muhammad Amin, Ahmadwali Aminee, Ireen Akhter Chowdhury, Kinley Dorji, Indrani Chakma, Aishath Shahula Ahmed, Hari Prasad Pokhrel, Danielle Schoenaker, Jane Hirst, Ranadip Chowdhury, Naomi M. Saville, Faith Miller, Zivai Murira, Vani Sethi

**Affiliations:** a Population Council Consulting, New Delhi, India; b Population Council, New Delhi, India; c United Nations Children's Fund (UNICEF), Sri Lanka; d United Nations Children's Fund (UNICEF), India; e United Nations Children's Fund (UNICEF), Nepal; f United Nations Children's Fund (UNICEF), Pakistan; g United Nations Children's Fund (UNICEF), Afghanistan; h United Nations Children's Fund (UNICEF), Bangladesh; i United Nations Children's Fund (UNICEF), Bhutan; j United Nations Children's Fund (UNICEF), Maldives; k Department of Public Health, Ministry of Health, Bhutan; l School of Human Development and Health, Faculty of Medicine, University of Southampton, Southampton, United Kingdom; m MRC Lifecourse Epidemiology Centre, University of Southampton, Southampton, United Kingdom; n NIHR Southampton Biomedical Research Centre, University of Southampton and University Hospital Southampton NHS Foundation Trust, Southampton, United Kingdom; o The George Institute for Global Health, School of Public Health, Imperial College London, United Kingdom; p Society for Applied Studies, New Delhi, India; q Institute for Global Health, University College London, London, United Kingdom; r United Nations Children's Fund (UNICEF) Regional Office for South Asia, Kathmandu, Nepal

**Keywords:** Preconception nutrition, Preconception care, Women's nutritional care and support, Policies and programs, South Asia

## Abstract

The health and health behaviours of women before conception significantly influence maternal and child health outcomes. Despite growing evidence supporting preconception nutrition care, data on the implementation of related policies and programmes remains limited. This paper reviews public policies and programmes delivering preconception nutrition interventions in eight South Asian countries, targeting married pre-pregnant women aged 15–49 years and identifies the systems bottlenecks in programme implementation. Most countries, except Sri Lanka, lack universal programmes for health and nutrition screening, provision of essential micronutrients, counselling on healthy eating and treatment for at-risk women. Even in countries, where supportive policies exist, implementation of comprehensive nutrition services for pre-pregnant women faces significant bottlenecks across six health system building blocks. Addressing these barriers is critical to improving intervention effectiveness, programme implementation, and informed decision-making. Further testing of a proposed comprehensive algorithm for preconception nutrition in diverse country contexts across South Asia is necessary.

## Introduction

The months to years before conception offer a unique opportunity to provide care and improve the health of future mothers and their babies. The nutritional status of women before pregnancy impacts the growth, development, and long-term health of their babies.^1^, ^2^, ^3^ In 2013, the World Health Organization (WHO) outlined recommendations for preconception care, including a focus on nutrition.^4^ Following this, the WHO South-East Asia Regional Office (SEARO) recommended two key intervention packages—one targeting adolescents’ healthy transitions and the other focusing on pre-pregnancy care for both married and unmarried adults—to be delivered through schools, health facilities and in community settings.^5^ Two recent papers have highlighted the burden of malnutrition among reproductive-aged women across South Asia,^6^ and evidence on the effect of preconception nutrition interventions on pregnancy and birth outcomes in South Asia.^7^

While the burden of malnutrition on women is reasonably well-documented^6^ and certain preconception nutrition interventions have been shown to improve pregnancy and birth outcomes,^7^, ^8^, ^9^ less is known about policies and programmes focusing on preconception nutrition in South Asia. Moreover, health system preparedness to adopt the interventions and systems bottlenecks that impede implementation of preconception programmes in South Asia have not been analysed to date. Hence, to advance understanding of the programme and policy landscape in the region, in this paper, we present a summary of maternal health and nutrition public policies and programmes delivering preconception nutrition interventions in eight South Asian countries; and an analysis of the systems bottlenecks for delivering these interventions.

## Methods

To find a common, specific definition and target group for preconception nutrition we referred to UNICEF's programme guidance on maternal nutrition which includes recommendations for preconception nutrition.^3^ We also reviewed published articles, guidelines, technical specifications suggested by UN agencies, and ministerial guidelines. Further, in 2023, we organized an expert group consultation. For this paper, preconception includes both the pre-pregnancy period for nulliparous women and inter-pregnancy intervals for multiparous women of reproductive age (WRA) 15–49 years.

**Fig. 1:**
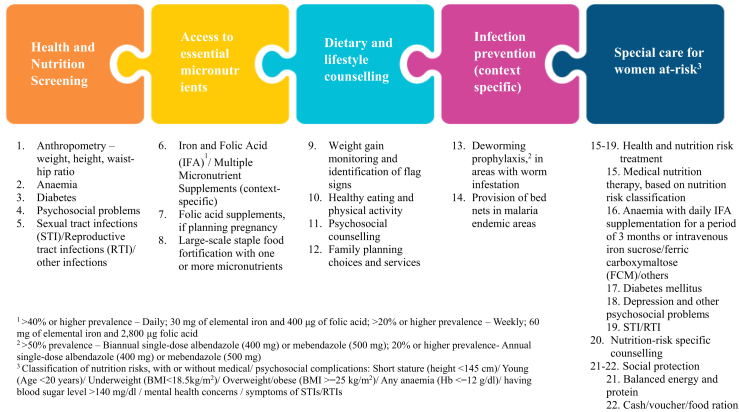
Five essential nutrition interventions for improving women’s nutritional care and support - before, between and beyond pregnancy.

Between August 2023 and July 2024, we followed four key steps, focusing on 15–49 year-old married preconception women across the eight South Asian countries–Afghanistan, Bangladesh, Bhutan, India, Maldives, Nepal, Pakistan, and Sri Lanka. Using the WHO recommendations on preconception care (2013) and UNICEF Programming Guidance on Maternal Nutrition (2022), 22 preconception nutrition interventions were categorized under five domains: (i) health and nutrition screening; (ii) access to essential micronutrients; (iii) dietary and lifestyle counselling; (iv) infection prevention; and (v) special care for women at risk (see Fig. 1).

We reviewed 118 national policies, legislation, and programme and implementation reports on preconception nutrition for each South Asian country using online searches (Supplementary Table S1). Subsequently, we held virtual consultations with UNICEF office staff from each of the eight countries to obtain additional relevant published and grey literature. We extracted data manually using a standardized template and mapped existing policies and programmes against each of the five intervention categories for the eight countries (Supplementary Table S2).

We then performed a systems bottleneck analysis for preconception nutrition interventions that were delivered through any programme. We developed a tool to assess health system bottlenecks in delivering preconception nutrition interventions based on similar bottlenecks analysis tools used for neonatal care, community health and assessments of health facility functionality in different low-income settings.^10^, ^11^, ^12^ We defined the recommended interventions for preconception nutrition by referring to WHO and UNICEF guidelines and mapped these against key health systems elements. Constraints to effective implementation were assessed according to existence of legislation and policies plus six parameters adapted from the WHO health system building blocks–(i) leadership, governance, and coordination; (ii) budget and financing; (iii) data and information systems, (iv) workforce; (v) essential commodities and supplies; and (vi) service delivery. We determined the level of intervention implementation bottlenecks in the health system using quantitative data and qualitative insights for the parameters mentioned above. Data from Demographic and Health Surveys provided quantitative indicators of intervention implementation. Qualitative insights were obtained through virtual discussions with country-level stakeholders including subject experts, policy makers, programme implementers, researchers, and UNICEF staff. Following similar bottlenecks analysis approaches used elsewhere,^10^, ^11^, ^12^ we assessed bottleneck severity using a systems bottleneck classification tool (Supplementary Table S3). For each health system component and each intervention, we computed an average across respondents’ bottleneck grading scores. The score for each parameter ranged between 0 and 2 with a higher score, representing lower severity. The severity was categorised into: no (score >1.5); mild (score 1.01–1.5); moderate (score 0.51–1.0); and significant (score ≤0.5) bottlenecks. The scoring system was simply used to identify the domains where the bottlenecks arose and which were most important within each country setting, rather than for a precise comparison of one county against another.

Finally, we conducted 43 stakeholder consultations across the 8 countries (between 3 and 11 per country) to validate the findings on the preconception nutrition programme and policy landscape and severity of bottlenecks in implementation of relevant programmes (Supplementary Table S4). UNICEF Regional Office for South Asia coordinated selection of stakeholders, prioritizing UNICEF personnel from each of the eight countries. Subsequently, country-specific UNICEF staff identified and recommended individuals with relevant expertise from both governmental and non-governmental sectors, including academic and subject matter experts, ensuring a diverse and knowledgeable group of stakeholders for consultation.

## Results

### Country specific preconception nutrition policies and programmes and associated system bottlenecks

A summary of the policies and programmes that include at least one component of preconception nutrition across the eight countries is presented in Table 1 (details are in Supplementary Table S2). Below we present the preconception nutrition interventions included in policies and programmes in the South Asian countries (Table 2) and associated system bottlenecks in implementing relevant programmes in each country, in alphabetical order.

**Table 1:** Summary listing of policies and programmes that include at least one component of preconception nutrition across the eight countries.

Countries	Number of policies that include at least one preconception nutrition interventions	Number of preconception interventions these policies cover	Programmes that include at least one preconception nutrition interventions	Number of preconception interventions these programmes cover	Number of interventions and programmes that cover WRA
Afghanistan	9 policies	20 interventions	5 programmes	9 interventions	6 interventions delivered through the 5 programmes
Bangladesh	19 policies	17 interventions	7 programmes	7 interventions	6 interventions delivered through 5 programmes
Bhutan	6 policies	19 interventions	6 programmes	19 interventions	12 interventions through 5 programmes
India	16 policies	20 interventions	10 programmes	18 interventions	9 interventions delivered through 6 programmes
Maldives	8 policies	15 interventions	3 programmes	13 interventions	4 interventions delivered through family planning programme and premarital session.
Nepal	19 policies	18 interventions	11 programmes	15 interventions	12 interventions delivered through 8 programmes
Pakistan	8 policies	18 interventions	6 programmes	7 interventions	4 interventions delivered through 6 programmes
Sri Lanka	10 policies	17 interventions	4 programmes	17 interventions	17 interventions through 4 programmes

**Table 2:** Preconception nutrition interventions included in policies and programmes in South Asia.




**Note:** NA- Not applicable due to low burden in the country.

^1^>40% or higher prevalence—Daily; 30–60 mg of elemental iron and 400 μg of folic acid; 20–39.9% prevalence—Weekly; 60 mg of elemental iron and 2800 μg folic acid.

^2^Underweight (BMI <18.5 kg/m^2^); Overweight/obese (BMI ≥ 25 kg/m2); Short stature (height <145 cm); Anaemia (Hb ≤ 12 g/dl); Diabetic (blood sugar level >140 mg/dl); Having mental health concerns; Having STI/RTIs.

^a^Universal program delivered through Upazila Health Complexes with no focus on pre-pregnant women.

^b^The policy only caters to adolescent girls 10–19 years.

^c^Intervention delivered through community clinics, with a primary focus on pregnant women; however, married and currently non-pregnant women are also covered.

^d^Policy and program both cater to tobacco and alcohol consumption. There is no mention of reducing caffeine.

^e^Only indicates counselling for STIs/RTIs and not treatment.

^f^The policy and programme cater to 10–19 years and 30 years and above age groups.

#### Afghanistan

In Afghanistan, 20 out of the 22 recommended preconception nutrition interventions are incorporated into 9 national policies. Of these 20 interventions, 9 are delivered through 5 programmes (Table 1). The policies include National Strategy for Prevention and Control of Non-Communicable Diseases (2015–2020),^13^ Mental Health Strategy (2019–2023),^14^ Regulations of Salt Iodisation (2011),^15^ Afghanistan Food Security and Nutrition Plan (2019–23),^16^ National Reproductive Health Policy (2012–2016),^17^ and National Malaria Strategic Plan, 2018–2022^18^ (Supplementary Table S2). Three policies – National Preconception Care Guideline (2022), National Reproductive, Maternal, Newborn, Child, and Adolescent Health Strategy (2024–2028), and Operational Guide for implementing the National Maternal, Infant and Young Child Nutrition (2019–2023) are grey literature. However, despite these policies, only 6 interventions have universal programmes that serve all WRA. These include screening and counselling for psychosocial problems delivered through the Basic Package of Health Services (BPHS),^19^ treatment for depression and other psychosocial issues through the Essential Package of Hospital Services^20^; provision of bed nets in malaria endemic areas through BPHS and Sehatmandi project^21^; large-scale food fortification via the universal salt iodization programme^22^; and family planning through BPHS^19^ and the National Family Planning Programme.

Some interventions currently have room for further development. To enhance the healthcare landscape, comprehensive programmes need to be expanded to include anthropometric assessments for women of childbearing age, and screening for anaemia, diabetes mellitus, sexually transmitted infections (STIs) or other reproductive tract infections (RTIs) (e.g., candidiasis, bacterial vaginosis), as well as iron folic acid (IFA) and folic acid supplementation, deworming prophylaxis, counselling on healthy eating and physical activity and reducing caffeine, alcohol, smoke, toxin exposure, balanced energy and protein (BEP) supplements and contextualised dietary modification. Additionally, policies and programmes in critical areas such as social protection interventions and medical nutrition therapy need strengthening.

We identified bottlenecks across six interventions included in the national programmes (Supplementary Table S5a). For screening and treating psychosocial issues, we found moderate bottlenecks in budget and financing, data and information systems, workforce, supplies, and service delivery. Psychosocial counselling faced moderate bottlenecks, similar to screening and treating psychological issues. For food fortification, we found moderate bottlenecks across all areas. Family planning interventions encountered moderate challenges in budget and financing. For malaria prevention, moderate bottlenecks were found in leadership and governance, budget and financing and supplies. Afghanistan's programme information system only captures data on 3 of the 6 universally implemented interventions: psychosocial counselling, family planning, and malaria prevention in endemic areas. Stakeholders highlighted that the ongoing humanitarian crisis critically hampers effective programme implementation.

#### Bangladesh

Bangladesh has 19 policies covering 17 of the 22 interventions and seven interventions are delivered through seven programmes (Table 1), including Bangladesh National Strategy for Maternal Health (2019–2030),^23^ Second National Plan of Action for Nutrition (2016–2025),^24^ National Strategy on Prevention and Control of Micronutrient Deficiencies (2015–2024),^25^ National Nutrition Policy, 2015,^26^ National Strategy for Adolescent Health (2017–2030),^27^ National Plan of Action for Adolescent Health Strategy (2017–2030),^28^ National Guideline on Diabetes Mellitus, 2023,^29^ National Protocol for Management of Diabetes and Hypertension (date not available),^30^ National Guidelines for Management of Sexually Transmitted Infections, 2020,^31^ National Strategic Plan for HIV and AIDS Response, 2018–2022,^32^ Mental Health Act, 2018,^33^ National Mental Health Strategic Plan (2020–2030),^34^ The Iodized Salt Act, 2021,^35^ Vitamin A Enrichment in Edible Oil Act, 2013,^36^ National Strategic Plan for Malaria Elimination in Bangladesh (2021–2025),^37^ and National Strategy for Anaemia Prevention and Control in Bangladesh, 2007^38^ (Supplementary Table S2). Two policies - National Nutrition Service Operational Plan, 2017–2022 and Universal Salt Iodization Strategy, 1989 are grey literature. Seven of these 17 interventions are implemented through nationwide government-funded programmes. The weekly IFA supplementation programme is only for adolescent girls aged 10–19 years through the National Nutrition Services Program,^44^ but the remaining six interventions have universal programmes catering to all WRA. These include folic acid supplementation for women planning pregnancies, counselling on healthy eating and reducing caffeine/alcohol/smoking through community clinics (primarily focused on pregnant women but also covers married non-pregnant women), large-scale fortification via the Universal Salt Iodization Program,^39^ family planning services through various health sector programmes,^39^^,^^40^ and provision of bed nets in malaria-endemic areas through the National Malaria Elimination Program.^37^

Despite this strong foundation, opportunities to further develop Bangladesh's policies and programmes include anthropometric assessment, social protection, BEP supplements, contextualized dietary counselling, and medical nutrition therapy. The availability of family planning services programme monitoring data highlights existing strengths and provides a solid basis for expanding and enhancing data for other areas of healthcare (Table 3).

**Table 3:** Data systems for preconception interventions in South Asia.




**Note:** NA- Not applicable due to low burden in the country.

^1^>40% or higher prevalence—Daily; 30–60 mg of elemental iron and 400 μg of folic acid; 20–39.9% prevalence—Weekly; 60 mg of elemental iron and 2800 μg folic acid.

^2^Underweight (BMI <18.5 kg/m^2^); Overweight/obese (BMI ≥ 25 kg/m2); Short stature (height <145 cm); Anaemia (Hb ≤ 12 g/dl); Diabetic (blood sugar level >140 mg/dl); Having mental health concerns; Having STI/RTIs.

x: Intervention not needed due to low burden in the country.

The bottleneck analysis for these six interventions revealed that five interventions (folic acid and IFA supplementation, large-scale food fortification, and counselling on healthy eating and reducing caffeine/alcohol/smoke/toxins exposure) faced moderate to significant bottlenecks in at least five components, including budget, data, workforce, supplies, and service delivery (Supplementary Table S5b). Family planning services encountered bottlenecks primarily in workforce, supplies, and service delivery.

#### Bhutan

Bhutan has six policies for WRA, covering 19 out of the 22 interventions and are delivered through six programmes (Table 1). Twelve of these 19 interventions are delivered to all WRA nationwide through government-funded programmes. These interventions include screening and treatment for anaemia, STIs/RTIs, and psychosocial issues, and counselling on healthy eating and physical activity, delivered through district-level health facilities and youth-friendly health centres. Other universal interventions include food fortification via the iodine deficiency control programme,^41^ family planning services through National Family Planning Programme and Youth Friendly Health Services,^42^ contextualised dietary counselling and medical nutrition therapy through all health facilities at district level, and bed net distribution through the National Malaria Control Program^43^ (Supplementary Table S2).

However, five interventions, such as folic acid and IFA supplementation, deworming, counselling to reduce caffeine/alcohol/smoke/toxin exposure, and psychosocial counselling, target married adolescent girls only and do not cover the entire WRA age range of 15–49 years. Additionally, screening and treatment for diabetes mellitus are provided only in select districts under the “Service with Care and Compassion Initiatives” based on WHO's Package of Essential NCDs (PEN).^44^ Bhutan has made significant strides in health policy, though there are opportunities to further expand policies and programmes in key areas such as anthropometric assessment, social protection, and BEP supplementation. Additionally, enhancing programme monitoring systems beyond family planning and malaria prevention, currently tracked through DHIS 2, could further strengthen Bhutan's healthcare delivery (Table 3).

We identified various bottlenecks across different nutrition interventions targeting pre-pregnant women. Data and information system challenges were found in screening and treatment for anaemia, diabetes mellitus, STIs/RTIs, psychosocial issues, and folic acid supplementation. Screening for psychosocial concerns, psychosocial counselling, deworming and counselling to reduce caffeine, alcohol, smoking, and toxin exposure faced moderate bottlenecks across all areas (Supplementary Table S5c). Additionally, moderate bottlenecks were noted in data systems for food fortification, and in most areas except governance for counselling on healthy eating and physical activity.

#### India

India has 16 policies that include 20 of the 22 interventions, with 18 interventions delivered through 10 programmes (Table 1). Of these, 9 interventions have universal coverage, including screening, treatment and counselling for STIs/RTIs, counselling on healthy eating and physical activity, and counselling to reduce caffeine/alcohol/smoking exposure, delivered through Village Health, Sanitation, and Nutrition Days (VHSNDs)^45^ and the Rashtriya Kishore Swasthya Karyakram (RKSK or National Adolescent Health Programme).^46^ Other interventions include screening for psychosocial problems and treatment and counselling of depression and other psychosocial issues through the RKSK^46^ and Ayushman Bharat Health and Wellness Centres.^47^ Large-scale food fortification is implemented through the Universal Salt Iodization Program and Rice Fortification Initiative, as part of Pradhan Mantri Garib Kalyan Anna Yojana (Prime Minister Food Welfare Scheme for Poor). Family planning services are provided under the National Health Mission (NHM) nationally and Mission Parivar Vikas (Family Development) Program in 7 states,^48^ RKSK,^46^ VHSND,^45^ and incentives to Accredited Social Health Activist (ASHA, female community-based health worker) through the NHM^49^ (Supplementary Table S2).

Nine out of 18 interventions have policies and/or programmes tailored to specific sub-target groups. These include anthropometric assessments and contextualized dietary counselling delivered to married adolescent girls through RKSK,^42^ anaemia screening in adolescent girls through the Anaemia Mukt Bharat (AMB, Anaemia free India) programme,^50^ diabetes screening and treatment through RKSK^46^ and National Programme for Prevention and Control of Non-Communicable Diseases,^51^ IFA supplementation and anaemia treatment through the AMB programme and RKSK, deworming prophylaxis through National Deworming Day (NDD)^52^ and RKSK,^46^ and nutrition and psychosocial counselling through RKSK, Ayushman Bharat Health and Wellness Centers, and the District Mental Health Program (DMHP)^50^ (Supplementary Table S2).

While India has established robust policies, opportunities to further enhance programme implementation exist. Folic acid supplementation and BEP supplementation for women in preconception age groups are areas for potential development. Additionally, expanding policies and programmes in social protection and medical nutrition therapy based on nutrition risk classification could strengthen the healthcare system. It is encouraging that programme service delivery/coverage data is available for IFA supplementation, family planning, deworming prophylaxis, anaemia and STI/RTI treatment through the Health Management Information System (HMIS) (Table 3),^53^ providing a solid foundation for future improvements.

We identified bottlenecks in all intervention areas for pre-pregnant women in India. Challenges include legislation and policies, leadership, governance, and coordination, budget and financing, data systems, workforce, essential supplies, and service delivery. Bottlenecks were prominent for anthropometric assessments, screening for diabetes mellitus, psychosocial problems, counselling on healthy eating and physical activity, reducing caffeine, alcohol, smoking/toxin exposure, dietary modification, and treatment of anaemia, diabetes, depression, and STIs/RTIs. Screening for anaemia faced bottlenecks in all areas except legislation and policies. Moderate bottlenecks were also noted in budget and financing, information systems, workforce, essential supplies, and service delivery, for IFA supplementation. Food fortification programmes encountered bottlenecks in leadership, management, governance, budget and financing, workforce, supplies, service delivery, and information systems. Family planning interventions faced challenges in leadership, governance, and coordination, and workforce, while deworming interventions had bottlenecks in legislation and policies, leadership, management and governance, budget and financing, data systems, and service delivery (Supplementary Table S5d).

#### Maldives

The Maldives has 8 national policies or guidelines for 15 out of 22 interventions, of which 13 interventions delivered through 3 programmes. Four interventions have programmes that cater to all WRA and nine targeted to specific subgroups (Table 1), such as adolescent girls and young women aged 10–24 years. Four interventions have universal coverage, including STI/RTI screening, counselling, and treatment through adolescent and youth-friendly health services^54^ and routine family planning visits, counselling on healthy eating and physical activity during premarital sessions, and family planning services through the National Family Planning Program.^55^ Two programmes target either a specific subgroup i.e., adolescent girls and young women aged 10–24 years or piloted in select atolls, offering services like anthropometric assessments; diabetes screening and treatment; IFA supplementation; and contextualized dietary counselling through the primary healthcare model^56^ and Adolescent and Youth Friendly Health Services^54^ (Supplementary Table S2). These two programmes also include screening, counselling and treatment for depression and other psychosocial problems and counselling aimed at reducing alcohol, caffeine, smoke, and toxin exposure.

Despite policies being established, challenges in programme implementation persist, particularly in food fortification and anaemia screening. Additionally, the Maldives lacks both policies and programmes in critical areas such as folic acid supplementation, deworming prophylaxis, social protection interventions, BEP supplements, medical nutrition therapy, and anaemia treatment. Program monitoring is currently available only for family planning through the HMIS (Table 3).

We identified bottlenecks across multiple components for various interventions, particularly for all five interventions under health and nutrition screening, IFA supplementation, counselling to reduce caffeine/alcohol/smoking, psychosocial counselling, contextualized dietary modification linked counselling, and treatment for diabetes, STI/RTI, and psychosocial problems. Family planning interventions faced challenges in budget and financing (Supplementary Table S5e).

#### Nepal

Nepal has 19 national policies or guidelines for 18 out of the 22 interventions, with these policies translating into 11 programmes for 15 interventions (Table 1). Of these, 12 interventions have universal programmes that serve all WRA. These include diabetes screening and treatment under the PEN,^57^ STI/RTI screening and treatment through the National HIV/AIDS and STI Control Programme, psychosocial problem screening, counselling and treatment via the primary healthcare system,^58^ folic acid supplementation for newly married women through newly married women programme, food fortification through universal salt iodization,^59^ and counselling on healthy eating and physical activity through the healthcare system and NCD prevention programme.^57^ Family planning services are offered through the national Family Planning Programme,^60^ and bed nets are provided in malaria-endemic areas under the National Malaria Control Programme.^61^ Three interventions have programmes tailored to specific subgroups or piloted in some provinces. These include provision of IFA supplements through the Weekly Iron and Folic Acid Supplementation Programme for out of school adolescent girls through facilities.^62^ Deworming for school-going adolescent girls aged 10–19 years and WRA are delivered through the School Health and Nutrition Programme^63^ and Female Community Health Volunteer Programme, respectively. Counselling to reduce caffeine, alcohol, smoking, and toxin exposure is through the National Tobacco Control Programme^64^ (Supplementary Table S2).

Despite these established policies, gaps remain in programme implementation, particularly in anthropometric assessment, anaemia screening and BEP supplementation. Nepal also lacks both policies and programmes in several critical areas, such as anaemia treatment, social protection, contextualized dietary counselling, and medical nutrition therapy. Programme monitoring data is available only for family planning and bed net provision through the HMIS (Table 3).

Bottlenecks were identified across the 15 interventions in Nepal. While most interventions face challenges across the six building blocks, family planning, food fortification, and malaria prevention interventions had fewer bottlenecks. Family planning, however, encountered challenges with data and information systems; food fortification faced challenges in leadership and governance, budgeting, and data systems; and malaria prevention struggled mainly with budget and financing (Supplementary Table S5f).

#### Pakistan

Pakistan has 8 policies that address 18 out of 22 interventions with 6 programmes delivering 7 interventions (Table 1). Four interventions are delivered through nationwide programmes for all WRA, while three are targeted to adolescents aged 15–19 years. The policies include the Maternal Nutrition Strategy (2022–27),^65^ National Guidelines for the Management of Sexually Transmitted Infections,^66^ Multi-sectoral Nutrition Strategy (2018–2025),^67^ National Integrated Reproductive, Maternal, Newborn, Child, Adolescent Health & Nutrition Strategy (2016–2020),^68^ Adolescent Nutrition Strategy and Operational Plan (2020),^69^ and the National Strategic Plan for Malaria Elimination (2021–2035)^70^ (Supplementary Table S2).

The nationwide programmes include weekly IFA supplementation for women planning pregnancy, food fortification through the Universal Salt Iodization Programme (established in 1994),^71^ family planning services through the National Programme for Family Planning and Primary Health Care,^72^ and mass distribution of long-lasting insecticide nets (LLINs) via the Malaria Eradication Programme. The three targeted programmes focus on STI/RTI screening and treatment, as well as the treatment of psychosocial issues, all delivered through the Essential Package of Health Services (EPHS).^73^

Programme implementation is lacking in key areas including anthropometric assessment, screening for anaemia, diabetes mellitus, and psychosocial issues, provision of folic acid, deworming prophylaxis, counselling on healthy eating and physical activity, counselling linked to contextualized dietary modification, BEP supplements, and treatment for anaemia and diabetes mellitus. Pakistan lacks both policies and programmes in counselling to reduce caffeine, alcohol, smoke and toxin exposure, psychosocial counselling, social protection, and medical nutrition therapy. Program monitoring data is available for IFA supplementation, provision of bed nets in malaria endemic areas, and family planning through the HMIS (Table 3).

We identified bottlenecks across the building blocks for the seven implemented interventions. Screening and treatment for STIs/RTIs and psychosocial problems face bottlenecks in all aspects. Food fortification intervention had moderate bottlenecks in data and information systems. Family planning had moderate challenges in leadership and governance, budget and financing, workforce, and service delivery (Supplementary Table S5g).

#### Sri Lanka

Sri Lanka has 10 policies and 4 programmes that deliver 17 out of 22 interventions (Table 1) for all WRA. Malaria prevention and deworming prophylaxis are not relevant for Sri Lanka, as the country was certified malaria-free by WHO in 2016,^74^ and the burden of worm infestation is low.^75^ The Service Package for Newly Married Couples, 2018,^76^ along with other policies, guidelines^77^, ^78^, ^79^, ^80^, ^81^, ^82^, ^83^, ^84^ and programmes^81^^,^^85^^,^^86^ (Supplementary Table S2) address anthropometric assessment, screening and treatment for anaemia, diabetes mellitus, STIs/RTIs, and psychosocial issues, folic acid supplementation, food fortification, and counselling on healthy eating, physical activity, counselling to reduce caffeine/alcohol/smoke/toxin exposure, psychosocial counselling, family planning, counselling linked to contextualized dietary modification and medical nutrition therapy.

However, preconception IFA supplementation, social protection, and BEP supplements, are neither mentioned in policies nor implemented through programmes. Program monitoring data is available only for folic acid supplementation and family planning services (Table 3).

We found implementation bottlenecks in a few interventions, but the rest had no challenges. Screening for anaemia and diabetes faced challenges with essential commodities and supplies, while screening for psychosocial concerns had bottlenecks in workforce, essential commodities and supplies, and service delivery. Furthermore, psychosocial counselling faced bottlenecks in almost all domains. Food fortification had data and information bottlenecks (Supplementary Table S5h).

#### Recommended actions against five specific strategies

Together with each UNICEF country team, we reviewed the programme and policy landscape and implementation bottlenecks and formulated action points aligned with five specific strategies to be taken up by the development partners, including UN agencies, non-governmental organizations, bilateral donors, and other relevant stakeholders (Table 4). We envision that these development partners should support governments to ensure that these actions are undertaken and own plans to sustain them.

1. **Science-drivenadvocacy for strengthened policies, financing and accountability**

**Table 4:** Specific actions for the development partners against the five strategies in the eight South Asian countries.

Strategies and actions	Afghanistan	Bangladesh	Bhutan	India	Maldives	Nepal	Pakistan	Sri Lanka
**Strategy 1: Science-drivenadvocacy for strengthened policies, financing and accountability**								
Depending on the country context, advocate with various stakeholders through technical, academic and policy consultations and dialogues								
a) for sensitization on the importance of preconception intervention	✓		✓		✓		✓	
b) to integrate within or update existing policies, programs or platforms		✓	✓	✓		✓	✓	
c) to strengthen/align with existing policies, programs or platforms								✓
**Strategy 2: Strengthen guidelines with costed plans and institutional architecture to improvedelivery of preconception nutrition services through food, health, social protection systems**								
Advocate with stakeholders to develop guidelines for preconception nutrition services and strengthen implementation through collaborations/models for effective coverage	✓	✓	–	✓	✓	✓	✓	✓
Support governments to address the identified systems bottlenecks for effective service delivery coverage through design, planning and leveraging domestic financing.		✓	✓		✓		✓	✓
Support governments to build capacity of research, academic and training institutions.			✓					
**Strategy 3: Increase the capacity and support to service providers at facility and community levels to deliver preconception nutrition services through community partnerships**								
Strengthen frontline health workers/service providers capacity through training, supervision, and SBC tools, working through local partners or pools of trainers and in convergence with other departments for delivering and monitoring preconception care services	✓	✓	✓	✓	✓	✓	✓	✓
Work with the stakeholders to develop/integrate/strengthen training modules for preconception care		✓		✓	✓		✓	✓
**Strategy 4: Increase supplies of essential commodities to prevent stockouts and enhance local production**								
Advocate with stakeholders to provide IFA/MMS in preconception			✓				✓	
Advocate with stakeholders to strengthen supply systems for supplements and equipment, based on demand forecasting and appropriate funding	✓	✓	✓	✓	✓	✓	✓	✓
**Strategy 5: Harness data and generate evidence in development and humanitarian settings via implementation research to inform policy and programme decisions**								
Support stakeholders to strengthen routine program reporting and monitoring systems towards preconception care interventions	✓	✓	✓	✓	✓	✓	✓	✓
Support in instituting periodic population-based surveys/real-time demonstrations on preconception interventions to generate evidence		✓	✓	✓		✓		✓

**Note:**Blank cells denote that this particular activity was not identified/proposed by the respective country stakeholders.

Sensitise stakeholders on the importance of preconception interventions through technical, academic and policy consultations, especially in countries where preconception nutrition pilots are lacking. Integrate preconception nutrition within the existing policies/programmes/platforms.

2. **Strengthening guidelines with costed plans and institutional architecture to improve delivery of preconception nutrition services through food, health, social protection systems**

Collaboratively develop and implement guidelines for preconception nutrition services, address system bottlenecks through design, planning, and domestic financing, and support institutional capacity building via research and training institutions.

3. **Increase the capacity and support to service providers at facility and community levels to deliver preconception nutrition services through and community partnerships**

Enhance frontline health workers' capacities through training, supervision, and social and behaviour change tools, and develop and integrate training modules for preconception care.

4. **Increase supplies of essential commoditiestoprevent stockouts and enhance local production**

Integrate IFA/Multiple Micronutrient Supplements (MMS) into preconception care and enhance supply systems through demand forecasting and adequate funding for supplements and equipment.

5. **Harness data and generate evidencein development and humanitarian settingsthrough implementation research to inform policy and programme decisions**

Enhance routine preconception care programme reporting and monitoring and generate evidence on preconception interventions through periodic population-based studies, surveys, and real-time demonstrations.

#### Proposed algorithm for women's nutritional care and support before, between and beyond pregnancy

**Fig. 2:**
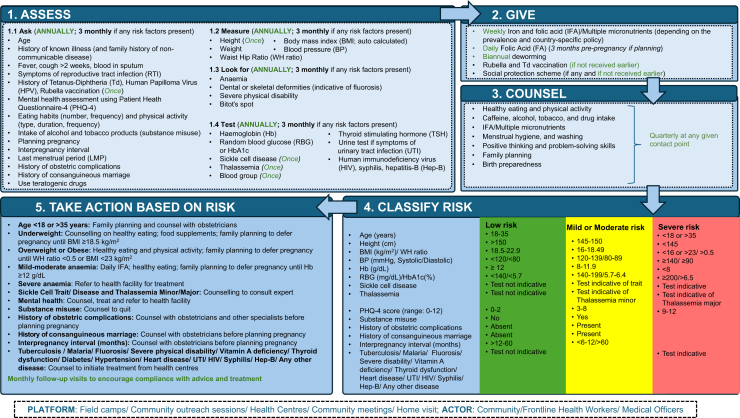
**Five Actions for women's nutritional care and support—before, between and beyond pregnancy**.

Based on a previously drafted algorithm by UNICEF India and Department of Health and Family Welfare India for preconception services delivered through village health sanitation and nutrition day, and reviewed program pathways from the various pilot interventions and programmes in different countries of South Asia, we propose an updated algorithm for women's nutritional care and support before, between and beyond pregnancies. This suggested algorithm illustrates 5 actions for all eligible women aged 15–49 years, married, non-pregnant and non-protected couple/women (Fig. 2). The five actions include (i) health and nutrition **assessment** (ask, measure, look for and test), (ii) **give**weekly IFA/MMS (depending on the prevalence and country-specific policy), daily folic acid supplements (if planning pregnancy), biannual deworming, and rubella and Tetanus-Diphtheria (Td) vaccination (if not received), and link to social protection schemes, (iii) **counsel** on healthy eating and physical activity; caffeine intake, alcohol, tobacco, and drug use; consumption of IFA/MMS; menstrual hygiene and hand washing; positive thinking and problem-solving skills; family planning; and birth preparedness; (iv) **risk classification** based on the health and nutritional assessment, and (v) **specific actions** for those women who are at health and nutritional risk. The possible platforms could be field camps, community outreach sessions, health centers, community meetings and home visit; while the actors could include frontline health workers and medical officers.

## Discussion

We present a comprehensive approach to mapping preconception nutrition policies, programmes, and data systems while identifying systemic implementation bottlenecks in eight South Asian countries. The policy and programme documents rarely explicitly define the preconception period; however, they mention WRA, specific sub-groups or newly married couples as the target population. Our findings indicate significant variability across the region in the implementation of evidence-based interventions. A major finding is the uneven coverage of preconception nutrition interventions. While almost all countries have integrated aspects of preconception nutrition into maternal health policies, only Sri Lanka has fully implemented preconception care as a national programme. Other countries have initiatives partially addressing preconception nutrition, targeting WRA or adolescent girls aged 10–19 years. This partial coverage underscores the critical need for explicit preconception frameworks to ensure universal access to essential interventions.

Some countries exhibit extensive policy frameworks; however, universal delivery of interventions is hampered due to bottlenecks in financing, workforce availability, and essential commodities for the preconception population. These findings align with global evidence suggesting that resource allocation and service delivery challenges persistently undermine the effectiveness of nutrition interventions in low- and middle-income countries.^87^^,^^88^ Limited budget allocation has been repeatedly documented as a critical impediment to nutritional interventions in the region, reaffirming our findings.^89^ Enhanced advocacy and domestic investment by government are crucial to addressing these financial barriers effectively. Furthermore, consistent with existing literature,^90^ inadequate training and workforce shortages were identified as major constraints, particularly at the community level, suggesting a clear need for targeted capacity-building and sustained workforce development.

We have proposed an algorithm of the five actions for women's nutritional care and support before, between and beyond pregnancy. However, feasibility of these proposed actions needs to be tested, at least in some of the South Asian countries with differing country contexts. Health and nutrition screening interventions, essential for early detection and prevention of adverse pregnancy outcomes,^2^^,^^91^ are inadequately implemented across the region. Although most countries have policies covering malnutrition, anaemia, diabetes, mental health, and STI/RTI screenings, comprehensive implementation is limited. Only Sri Lanka, Bhutan, and Nepal offer relatively comprehensive programmes. These findings resonate with global evidence emphasizing the benefits of early identification and treatment of nutritional and health risks to enhance maternal and child outcomes.^92^^,^^93^

Essential micronutrients, such as folic acid and IFA supplementation to adolescent girls, remain inconsistent despite universal policy frameworks. Bangladesh, Nepal, and Sri Lanka provide preconception folic acid supplements, while large-scale food fortification efforts, predominantly iodized salt, lack routine monitoring except in Nepal. Strengthening implementation and monitoring of micronutrient programmes remains crucial, given the well-documented benefits in reducing birth defects and improving maternal nutrition status.^93^

Most South Asian countries have dietary and lifestyle counselling policies; however, comprehensive universal implementation remains limited. Notably, Sri Lanka, Bangladesh, and India have achieved extensive coverage, addressing harmful exposures like alcohol, tobacco, and environmental toxins, aligning with global recommendations on reducing modifiable risk factors.^87^ Nonetheless, broader regional efforts to ensure universal counselling access are required.

Infection prevention interventions, specifically for malaria and worm infestations, remain fragmented. While bed net provision in malaria-endemic regions is universally recognized as crucial, coverage is incomplete. Deworming policies exhibit considerable variability, often targeting only adolescent girls or specific sub-populations, despite the known link between infections, anaemia, and adverse maternal and neonatal outcomes.^94^^,^^95^

Comprehensive care for women at risk remains largely undeveloped across South Asia, despite the high prevalence of unplanned pregnancies. Only Sri Lanka demonstrates effective universal management of nutritional and health risks before pregnancy. The absence of comprehensive policies for nutritional management, social protection, and targeted interventions for at-risk women represents a significant missed opportunity for improving maternal and child health outcomes.^96^

Data and monitoring systems across the region are weak and fragmented. Reliable survey and routine monitoring data for preconception nutrition interventions are notably scarce, severely restricting evidence-based policymaking and programme refinement.^97^ Strengthening these systems, as observed through the robust monitoring frameworks in Sri Lanka and select interventions in Nepal and India, can significantly improve outcomes and policy responsiveness.

Our findings suggest several strategic recommendations: firstly, explicit integration of comprehensive nutrition interventions for preconception population within national policies; secondly, prioritizing resource allocation and strengthening health system capacities, particularly in workforce development; thirdly, investing in robust data and monitoring frameworks; and finally, adopting multi-sectoral approaches incorporating health, education, and social protection sectors to enhance sustainability.

Our study's strength is that it combines a comprehensive review of policies and programmes, bottleneck analysis of programme implementation, with insights from experts and decision-makers. This provided robust and validated findings to inform recommended actions. However, the study had some limitations. The bottleneck analysis was conducted at the national level, which may limit the applicability of findings at sub-national levels. The tool asked 31 questions for each of the five domains of preconception nutrition intervention, which may have generated response bias due to respondent fatigue. Further, the reported bottlenecks we identified were perceived bottlenecks which warrant triangulation against other sources. Country-level stakeholder discussions varied in format, which may have led to differences in how priorities were addressed across countries. Lastly, it was not possible to assess quality and coverage of implementation. However, our findings form a useful basis for field studies to investigate quality and coverage.

## Conclusion

Policy makers and programme implementers must prioritize targeted and coordinated interventions for preconception women to achieve both the immediate and sustained benefits of preconception nutrition. The benefits need to be looked from the women's health and well-being perspective, not merely to improve future pregnancy outcomes. While many South Asian countries have established policy frameworks, significant challenges persist in translating these policies into effective programme implementation. Lack of programme data impedes decision-making and limits understanding of intervention effectiveness. The presence of moderate to significant bottlenecks highlights the urgent need for enhanced programme management and resource mobilization.

Key actions to overcome these barriers include systematically scaling up comprehensive nutrition intervention packages tailored for pre-pregnant women, ensuring robust and routine collection of programme data to support informed decision-making, and strengthening legal frameworks along with rigorous enforcement mechanisms to monitor and improve the nutritional food environment. Additionally, establishing clear accountability structures and performance metrics will help track progress and enhance intervention effectiveness.

Multilateral organizations, national and sub-national governments, academia, health systems, and civil society must form cohesive, multi-sectoral partnerships with clearly defined roles and commitments. By collectively addressing identified challenges and implementing these strategic actions, stakeholders can significantly advance the nutritional health and wellbeing of women before, between, and beyond pregnancy.

## Contributors

VS, ZM and AH conceived the study. AH, MS and RV designed the protocol and VS provided inputs. TC, MS and CG conducted literature searches. RV and AH reviewed the secondary data. MS, AH, RV and VS designed the system bottleneck analysis tool. TC, MS, RV, CG and AH conducted stakeholder consultation for obtaining country specific documents, inputs on the system bottleneck analysis and validation of the results. DR, AD, PM, NP, NA, MdS, WK, KW, MA, IAC, KD, AA, IC and AS supported in the process of stakeholder consultation and provided input on the findings and validation of the results. TC and MS helped in revising the tables, figures and drafts. All authors read and commented on various drafts and approved the final manuscript.

## Data sharing statement

We have used secondary information available in the public domain. Data extraction tables can be made available by request.

## Declaration of interests

This work was funded by UNICEF Regional Office for South Asia (ROSA) contract number 43386162.

The funder, UNICEF Regional Office of South Asia, was involved in the design of the review, provided oversight in its conduct and are coauthors (ZM, VS). The corresponding author had full access to all the data and the final responsibility to submit it for publication.

Co-authors ZM and VS are employees of UNICEF Regional Office for south Asia which funded this study. Other authors have no conflicts of interest. The authors declare no other conflicts of interest.

The statements in this publication are the views of the author(s) and do not necessarily reflect the policies or the views of UNICEF. The designations employed in this publication and the presentation of the material do not imply on the part of the UNICEF the expression of any opinion whatsoever concerning the legal status of any country or area, or of its authorities or the delimitations of its frontiers.
