# Supplementary material for: Policies and programmes to improve preconception nutrition in South Asia
Source: Lancet Reg Health Southeast Asia. 2025 May 1;36:100589. doi: 10.1016/j.lansea.2025.100589 (PMC12105511; doi:10.1016/j.lansea.2025.100589)
Supplement: Supplementary Tables [file mmc1.docx]

**Supplementary Tables**

**S1. Details of the search process**

**Defined types of documents to be reviewed**

- Policies
- Legislations
- Program guidelines/strategies
- Implementation plans
- Advocacy & communication materials
- Training materials
- Monitoring, recording & reporting formats

**Identified database/ source**

- Google search
- Google scholar
- PubMed
- Government ministerial websites
- Grey literature from UNICEF country offices

**Defined search terms and period**

- Search terms*
- Search period: October 2023 to May 2024

**Retrieved relevant documents** (N=180)

- Afghanistan: N=21
- Bangladesh: N=26
- Bhutan: N=15
- India: N=32
- Maldives: N=15
- Nepal: N=35
- Pakistan: N=19
- Sri Lanka: N=17

**Reviewed and evidence synthesized**

(N=118)

- Afghanistan: N=15
- Bangladesh: N=22
- Bhutan: N=8
- India: N=23
- Maldives: N=9
- Nepal: N=18
- Pakistan: N=12
- Sri Lanka: N=11

**Excluded** (N=62)

- Latest version of same document used for the review
- Document did not include non-pregnant target population
- Not a policy or strategy document
- Document did not cover relevant interventions

*(maternal OR pregnancy OR antenatal) AND (nutrition) AND (policies OR programs OR programme guidance OR strategies OR plans OR action plans OR guidelines OR training modules) AND (Country Name) (preconception OR pre-conception OR pre-pregnancy OR pre-pregnancy OR before pregnancy OR newly married OR pre marriage OR pre-marriage OR premarital OR pre-marital) AND (nutrition) AND (policies OR programs Or programme guidance OR strategies OR plans OR action plans OR guidelines OR training modules) AND (Country Name)

**S2. Details of the policies and program referred during the review for the eight South Asian countries**

| **Interventions** | **Afghanistan** | | **Bangladesh** | | **Bhutan** | | **India** | |
| --- | --- | --- | --- | --- | --- | --- | --- | --- |
|  | **Policy** | **Program** | **Policy** | **Program** | **Policy** | **Program** | **Policy** | **Program** |
| **I. Health and Nutrition Assessment** | | | | | | | | |
| Anthropometry (height, weight, or waist to hip ratio) | Yes  **National Preconception Care​Guideline, 2022:** Aims to monitor nutritional status. | No | No | No | No | No | Yes (partially)  **Rashtriya Kishor Swasthya Karyakram (RKSK) Operational Framework, 2014:** Covers BMI screening during Adolescent Health Days. | Yes (partially)  **RKSK:** Covers anthropometric screening of adolescents. |
| Anaemia (Blood test such as haemoglobin) | Yes  **National Preconception Care​ Guideline, 2022:** Covers anaemia screening. | No | Yes  **Bangladesh**  **National Strategy for**  **Maternal Health 2019-2030​:** Recommends screening for risk conditions such as anaemia and rubella. | No | Yes  **National Nutrition Strategy and Action Plan 2021-2025**: Covers screening and management of anaemia among WRA during outreach camps  **Youth Friendly Health Services Guide and Implementation Standards, 2008:** Aims to screen married adolescent girls and young people (10-24 years) for anaemia by providing routine haemoglobin estimation at healthcare facilities. | Yes  Universally provided through health facilities at the districts level  **Youth Friendly Health Services:**  Aims to provide Hb estimation for young people aged 10-24 years at BHU2 Facilities and through outreach clinics. | Yes  **RKSK Operational Framework, 2014:** Covers anaemia screening during Adolescent Health Days.  **Anaemia Mukt (Free) Bharat (AMB) Operational Guidelines, 2018:** Aims to encourage newlyweds and married women aged 20-24 years to undergo haemoglobin testing at the nearest health facility. | Yes (partially)  **AMB Programme:** Aims to screen adolescents (10-19 years) for anaemia in their communities during Adolescent Health Days. |
| Diabetes mellitus using oral glucose tolerance test | Yes  **National Strategy for Prevention and Control of Non-Communicable Diseases 2015-2020:** Covers the integration of NCDs such  as prevention and control of cardiovascular diseases, diabetes, and chronic pulmonary diseases in BPHS and EPHS.  **National Preconception Care Guideline, 2022:​** Covers screening for diabetes. | No | Yes  **National Guideline on Diabetes Mellitus, 2023:**  Recommends testing for prediabetes/diabetes to all women planning pregnancy.  **National Protocol for Management of Diabetes and Hypertension** (published date not available)**:** Covers screening for diabetes. | No  Universal delivery of programmes through Upazila Health Complexes with no focus on preconception women | **Yes**  **Bhutan Every Newborn Action Plan, 2016-2023:** Covers diagnosis and treatment of hypertension, diabetes, and hypothyroidism. | Yes (partially)  **Service with Care and Compassion** **Initiatives:** Based on the WHO Package of Essential NCDs (PEN)​ | Yes (partially)  **RKSK Operational Framework, 2014:** Covers screening for diabetes of out-of-school adolescent girls and boys aged 10-19 years at Adolescent Friendly Health Clinics (AFHCs)  **Ayushman Bharat, Health and Wellness Centres,**  **Operational Guidelines, 2018:** Recommends screening for diabetes, hypertension, and mental health conditions for all individuals above 30 years of age.  **Prevention, Screening, and Control of Non-Communicable Diseases (HTN, Diabetes, and Common Cancers), Operational Guidelines, 2016**: Covers annual diabetes screening for women aged 30 years and above through outreach services at the village level. | Yes (partially)  **RKSK and National**  **Programme for Prevention and Control of Non-Communicable Diseases:** Aims to provide screening and management of diabetes and hypertension. |
| Sexually transmitted infections (STIs) or reproductive tract infections (RTIs) | Yes  **National Reproductive, Maternal, Newborn, Child, and Adolescent Health Strategy 2024-2028:** Recommends the integration of syndromic management and primary prevention of STIs into the BPHS.  **National Preconception Care​**  **Guideline, 2022:** Covers screening for STIs. | No | Yes  **National Guidelines for Management of Sexually Transmitted Infections, 2020:** Covers STI screening.  **National Strategic Plan for HIV and AIDS Response 2018-2022:** Covers STI screening. | No  Universal delivery of programmes through Upazila Health Complexes with no focus on preconception women | Yes  **Bhutan Every Newborn Action Plan, 2016-2023:** Covers prevention and treatment of STIs/HIV. | Yes  Universally provided through health facilities at the districts level  **Youth Friendly Health Services:** Provides STI screening to adolescents aged 10-24 years through community and BHU2/outreach clinics. | Yes  **National Guidelines for Village Health, Sanitation & Nutrition Day (VHSND), 2019:** Provides screening for RTI/STI, including HIV/AIDS, to women aged 15-49 years, with referral to ICTC while ensuring confidentiality.  **RKSK Operational Framework, 2014**: Covers the identification of symptoms of RTI/STI during Adolescent Health Days using a syndromic approach. | Yes  **VHSND and RKSK:** Aim to provide screening for RTI/STI, including HIV/AIDS, to women aged 15-49 years, with referral to ICTC while ensuring confidentiality. |
| Psychosocial problems | Yes  **Mental Health Strategy 2019-2023 and National Preconception Care​**  **Guideline, 2022:** Covers assessment and provision of educational and psychosocial counselling before, during pregnancy and in the post-partum period. | Yes  **Basic Package of Health Services for Afghanistan:** Provides mental health education and awareness, as well as case identification, diagnosis, and treatment. | Yes  **Mental Health Act 2018; Mental Health Policy 2019; National Mental Health Strategic Plan 2020-2030**: In 2018, Bangladesh Parliament approved a new Mental Health Act. A new Mental Health Policy, approved by the Ministry of Health in 2019, reflected a shift from a medical to a psychosocial treatment model with emphasis on decentralization and community-based services and support for persons living with mental illness.  **National Strategy for Adolescent Health 2017-2030:** Promotes school and facility level interventions which include counselling and management of mental health disorders through linkage with the national mental health programme. | No  Universal delivery of programmes through Upazila Health Complexes with no focus on preconception women | Yes  **Bhutan Every Newborn Action Plan, 2016-2023:** Covers mental health programme as one of the interventions. | Yes  Universally provided through health facilities at the districts level  **Youth Friendly Health Centres:** Covers mental health interventions for adolescents aged 10-24 years, which include recognition of mental health problems in the community and referral for reporting, diagnosis and treatment services at the facility level, as well as awareness-raising on mental health at the community level. | Yes  **RKSK Operational Framework, 2014:** Covers the identification of adolescents with chronic/severe mental health issues for referral to AFHCs.  **National Mental Health Policy, 2014:** Aims to provide and increase access to and utilization of comprehensive mental health services including prevention, treatment and care services.  **Ayushman Bharat Health and Wellness Centres Operational Guidelines, 2018:​** Covers screening and basic management of mental health ailments (Psychosis, Depression, Neurosis, Dementia, Mental Retardation, Autism, Epilepsy and Substance Abuse related disorders). | Yes  **RKSK; Ayushman Bharat, Health and Wellness Centres:** Cover screening and basic management of mental health ailments (Psychosis, Depression, Neurosis, Dementia, Mental Retardation, Autism, Epilepsy and Substance Abuse related disorders) at the community level and through health and wellness centres. |
| **II. Provision of essential micronutrients** | | | | | | | | |
| Folic acid supplements, if planning pregnancy | Yes  **National Preconception Care​**  **Guideline, 2022**: Provides supplementation with iron, folic acid, and calcium. | No | Yes  **Bangladesh National Strategy for Maternal Health 2019-2030:**  Recommends the provision of iron and folate supplementation and deworming to adolescent and pre-pregnant women. | Yes (universal)  Provision of folic acid to all women who are planning pregnancy through community clinics.​. | Yes  **National Nutrition Strategy and Action Plan 2021-2025:** Covers folic acid supplementation. | Yes (partially)  Provision of folic acid to women who are planning pregnancy in few provinces. | Yes  **Strategic Approach to Reproductive, Maternal, Newborn, Child and Adolescent Health (RMNCH+A), 2013:** Recommends preventive use of peri-conception folic acid 3 months before and after conception  **India Newborn Action Plan, 2014**: Covers peri-conceptional folic acid.  **AMB Operational Guidelines, 2018:** Recommends the initiation of folic acid supplementation (400 mcg of folic acid tablets daily) to women planning pregnancy. | No |
| Weekly or daily IFA supplementation | Yes  **National Preconception Care​**  **Guideline, 2022:** Covers IFA supplementation.  **Operational Guide for Implementing the National Maternal, Infant and Young Child Nutrition (2019-2023):** Recommends universal IFA supplementation for adolescent girls 10-19 years and WRA with prevalence of severe anaemia ≥40%. | No | Yes  **National Strategy on Prevention and Control of Micronutrient Deficiencies 2015-24:** Recommends IFA provision to newlywed women and weekly IFA to NPNL adolescent girls aged 10-19 years through schools and adolescent community clubs.    **Bangladesh National Strategy for Maternal Health 2019-2030:**  Covers IFA and de-worming during adolescence and pre-pregnancy period.  **National Strategy for Adolescent Health 2017-2030:** Recommends including nutritional supplements such as iron folate in the essential medicines list.  **National Nutrition Policy, 2015:** Recommends strengthening nutrition specific interventions such as IFA supplementation for women and adolescent girls. | Yes (partially)  **National Nutrition Services, 2017-22:** Provides weekly IFA supplements to adolescent girls aged 10-19 years through schools and adolescent community clubs. | Yes  **Bhutan Every Newborn Action Plan, 2016-2023**: Recommends IFA supplementation for WRA as one of the interventions. | Yes (partially)  **Youth Friendly Health Services:** Provides weekly IFA to out of school adolescent boys and girls. | Yes  **India Newborn Action Plan, 2014:** Covers multiple micronutrient supplementation (IFA & Iodine) as one of the preconception interventions.  **AMB Operational Guidelines, 2018**; **RKSK Operational Framework, 2014:** Recommends weekly one tablet containing 60 mg elemental iron + 500 mcg folic acid for school-going adolescents 10-19 years and out-of-school adolescent girls 10–19 years, as well as for non-pregnant or non-lactating women 20-49 years. | Yes (partially)  **AMB programme:** Provides weekly tablets containing 60 mg elemental iron + 500 mcg folic acid to school going and out-of-school adolescents aged 10-19 years and non-pregnant women/women in reproductive age since 2023. Selected states have implementation guidelines with his provisions and have budgeted for procurement of IFA tablets for WRA women aged 20-24 years in selected states for 2024-26. |
| Large-scale food fortification of staple foods | Yes  **Regulations on Iodizing Salt​,**  **March 2011:** Covers salt iodization regulations under Article 33**​**  of the Health Law.  **Afghanistan Food Security and Nutrition Plan, 2019-23:** Aims to reduce micronutrient deficiencies (Vitamins A and D, iron, folic acid, iodine) by strengthening regulations and promoting the consumption of fortified food with special focus on iodised salt, fortified wheat and oil. | Yes  **Universal Salt Iodization Program:** Provides iodized salt.**​** | Yes  **National Strategy on Prevention and Control of Micronutrient Deficiencies, Bangladesh (2015-2024)**  **Universal Salt Iodization Strategy, 1989; Iodised Salt Act, 2021;** Vitamin A enrichment in Edible Oil Act, 2013  **National Strategy for Adolescent Health 2017-2030**  **National Plan of Action for Adolescent Health Strategy 2017-2030**  **National Plan of Action on Nutrition 2016-2025​** | Yes  **Universal Salt Iodization Program, Rice Fortification, Oil Fortification law with Vitamin A**  **National Nutrition Services Program, 2017-22:**  Recommends developing communication materials and conducting SBCC activities to promote the use of iodized salt and providing technical support to Ministry of Industries in monitoring and quality assessment. | Yes  **National Health Promotion Strategic Plan 2015-2023; National Nutrition Strategy and Action Plan 2021-2025:** Includes working with relevant government departments and the corporate sector to provide economic incentives to increase self-sufficiency for varieties of healthy foods and fortification of food with micronutrients​. | Yes  **IDD Control Programme:** Covers salt iodization. | Yes  **National Nutrition Policy, 1993:** Covers fortification of essential food items with appropriate nutrients (salt etc.), intensification of research in iron fortification of rice and other cereals, distribution of iodized salt to cover all the population groups in endemic areas of the country to reduce iodine deficiency to below endemic levels.  **Food Safety and Standards (Prohibition and Restriction on Sales) Regulations, 2011:** Covers regulations on sale of only iodized salt permitted for direct human consumption.  **Food Safety and Standards (Fortification of Foods) Regulations, 2018:** Provides mandatory fortification standards for staple foods that include rice, wheat flour, oil, salt, milk with vitamins and minerals if they are to be marketed and sold. | Yes  Mandatory iodization of salt since 1997. |
| **III. Counselling on healthy eating and family planning choices and services** | | | | | | | | |
| Counselling on healthy eating and physical activity | Yes  **Afghanistan Food Security and Nutrition Plan 2019-2023:** Mentions improving maternal (both pre-pregnancy and during pregnancy and lactation) and adolescent nutrition by promoting balanced and micronutrient rich diets and good nutrition practices, specific to women and adolescents girls using food based dietary guidelines broadly through the scale-up and strengthening of the nutrition components of BPHS, the EPHS, and other programs that provide opportunities for public communication and interpersonal counselling.  **Operational Guide for implementing the National Maternal, Infant and Young Child Nutrition (2019-2023):** Mentions conducting nutrition education and counselling on healthy practices to promote improved nutritional status and to reach married adolescents in particular (mentioned in the job aid).  **National Preconception Care Guideline, 2022:**​ mentions information, education and counselling and promoting exercise. | No | Yes  **Bangladesh National Strategy for Maternal Health, 2019-30:** Mentions pre-pregnancy counselling.    **Second National Plan of Action for Nutrition (2016-2025):** Prioritizes promotion of adolescent nutrition and healthy lifestyle through formal and informal academic curricula and training programs. Health seeking behaviour of adolescents, young/teenage couples are planned to be enhanced through facility and community-based approaches.  **National Strategy for Adolescent Health 2017-2030**: Provides community-based intervention for out of school adolescents, including targeted programme such as nutrition education and supplements during home visit and through adolescent clubs  **National Plan of Action for Adolescent Health Strategy 2017-2030**  **National Nutrition Service Operational Plan, 2017-2022** | Yes  **National Nutrition Services, 2017-22:** Provides counselling services through community clinics/meetings with a primary focus on pregnant women, married and currently non-pregnant women are covered too​. | Yes  **Bhutan Every Newborn Action Plan, 2016-2023**: Suggests counselling on nutrition to adolescent girls and WRA as one of the preconception interventions.  **National Nutrition Strategy and Action Plan 2021-2025:** Mentions improving counselling services on nutritious and safe diets, physical activity, rest and weight gain.  **Youth Friendly Health Services Guide and Implementation Standards, 2008:** Recommends service providers to provide information on balanced diet and consumption of green leafy vegetables and other iron rich foods. Nutrition education on balanced diet, sign and symptoms of common nutritional deficiencies like anaemia. | Yes  Universal through all health facilities at districts level.  **Youth friendly Health Service:​**Delivers nutrition counselling to married adolescents and young people (10-24 years). | Yes  **National Guidelines for VHSND, 2019:** Recommends group counselling to WRA (15-49 yrs) and family members on balanced diet, importance of dietary diversity for health and development, healthy food habits, IFA, and iron rich foods etc.  **RKSK Implementation Guidelines, 2015 :** Covers training manual for peer educators​. | Yes  **VHSND:** Covers group counselling, awareness generation and nutrition and Health education to WRA (15-49 years and family members) on balanced diet, importance of dietary diversity for health and development, locally drown nutrient rich crops, healthy food habits, hygienic and correct cooking practices, anaemia – causes and prevention as per AMB guidelines, iron rich foods, iron fortified foods and prevention of worm infestation and WASH.  **RKSK:** Includes nutrition education through outreach activities such as peer education programme and Adolescent Health Day. |
| Reduce caffeine/ alcohol/ smoke/ toxin exposure | Yes  **National Preconception Care Guideline, 2022:** Recommends screening of women and girls for tobacco use  at all clinical visits  and providing brief tobacco cessation advice, pharmacotherapy and intensive behavioural counselling services along with advising all men and women about harm of second-hand smoke and harmful effects on pregnant women and unborn children. | No | Yes  **Bangladesh National Strategy for Maternal Health, 2019-30​** | Yes  Delivered through community clinics/meetings with a primary focus on pregnant women, however married and currently non-pregnant women are covered too. | Yes  **Bhutan Every Newborn Action Plan, 2016-2023**  **Youth Friendly Health Services Guide and Implementation Standards, 2008** | Yes (partially)  **Youth friendly Health Service**​ | Yes  **National Guidelines for VHSND, 2019:** Includes counselling on NCD prevention includes avoidance of alcohol/tobacco cessation to mother of children (0-5 yrs), adolescent, women in reproductive age group specially newly married, and school children.  **RKSK operational framework, 2014** recommends awareness of adverse effects and consequences of tobacco, alcohol and drugs taken up during Adolescent Health Day and Kishori Samuhas (adolescent groups) under scheme for adolescent girls (SAGs)​ | Yes  **VHSND:** Includes counselling on NCD prevention includes avoidance of alcohol/tobacco cessation to mother of children (0-5 yrs), adolescent, women in reproductive age group esp. newly married, School Children specially newly married, and school children |
| Psychosocial counselling | Yes  **Mental Health Strategy 2019-2023; National Preconception Care Guideline, 2022:** Mentions providing educational and psychosocial counselling before, during  pregnancy and post-partum period. | Yes  **Basic Package of Health Services​:** Mentions the provision of psychosocial counselling and the HR and funds for the same. | Yes (partially)    **National Strategy for Adolescent Health 2017-2030; National Plan of Action for Adolescent Health Strategy 2017-2030:**  Mentions enabling evidence-based advocacy for comprehensive programme development to promote mental health among adolescents and reduce stigma against mental ill health; Develop skills among adolescents to deal with stress, manage conflict and develop healthy relationships. | No | Yes  **Bhutan Every Newborn Action Plan, 2016-2023:** Specifies to organize participation in de-addiction and smoking cessation programmes  **Youth Friendly Health Services Guide and Implementation Standards, 2008​:** Recommend identification, information and counselling related to alcohol and other substance abuse | Yes (partially)    **Youth Friendly Health Services** | Yes (partially)  **RKSK operational framework, 2014:** mentions provision of psychosocial counselling through Adolescent Friendly Health Clinics.  **National Mental Health Policy, 2014:** Aims to provide and increase access to mental health care and utilization of comprehensive mental health services including prevention services, treatment and care services.  **Ayushman Bharat Health and Wellness Centres Operational Guidelines, 2018:​** Includes screening and basic management of mental health ailments (Psychosis, Depression, Neurosis, Dementia, Mental Retardation, Autism, Epilepsy and Substance Abuse related disorders) | Yes (partially)  **RKSK and Ayushman Bharat Programme**    **District Mental Health Program (DMHP), 1996:** Includes interventions at the Community Health Centre and Primary Health Centre levels that include outpatient services, assessment, counselling/ psycho-social interventions, continuing care and support to persons with severe mental disorders, drugs, outreach services, ambulance services etc. |
| Family planning choices for delaying age at first pregnancy (>=20 years) / increase inter-birth interval (at least 24 months) | Yes  **Operational Guide for implementing the National Maternal, Infant and Young Child Nutrition (2019-2023)​; National Reproductive Health Policy 2012-2016; National Reproductive Health Strategy 2012-2016; National Preconception Care​** **Guideline, 2022**: Documents mention delaying of the first birth and adequate birth spacing.  high- parity pregnancies. | Yes  **Basic Package of Health Services; National Family Planning Program:** Covers counselling on and methods for birth spacing are given to women in the postpartum period under BPHS. | Yes  **Bangladesh National Strategy for Maternal Health 2019-2030:**  Covers couple  registration,  pre-pregnancy  counselling, and  birth spacing.  **National Nutrition Policy 2017-2022; National strategy for Adolescent Health 2017-2030; National Plan of Action for Adolescent Health Strategy 2017-2030:** Recommends registering newly married adolescent girls and adolescent mothers to reach them with FP counselling and services to delay the first pregnancy. Also, encourage to delay first pregnancy among newlywed adolescent girls by providing a gift pack containing OCP and condoms. | Yes  **Health, Nutrition, Population Sector Programme; Adolescent Friendly Health Corners; Family Planning Programme for Newly-Wed Couples** | Yes  **Bhutan Every Newborn Action Plan, 2016-2023:** Includes intervention regarding family planning such as counselling services to all women of reproductive age group to delay first child and ensure proper birth spacing and access to family planning service (if needed).  **National family planning standards, 2018**  **Youth Friendly Health Services Guide and Implementation Standards​, 2008:** Recommends counselling and provision of reversible contraceptives. | Yes  **National Family Planning Programme;** **Youth Friendly Health Services: ​**Provides available contraceptive choices for birth spacing such as long acting reversible contraceptive, short acting reversible contraceptive, etc. that are voluntary and free of charge. | Yes  **Mission Parivar Vikas Guidelines, 2016:** Includes newlywed couples  **National Guidelines for VHSND, 2019:** Includes counselling on spacing and limiting methods; where to access services; beneficiary incentives to women in reproductive age group specially newly married. | Yes  **Mission Parivar Vikas Program, 2017:** Provides “NAYI PAHEL”, a family planning kit to newlywed couples by ASHA in rural areas in MoHFW’s seven high fertility states  **RKSK program:** Includes counselling of newly married couples on the risks of early conception and the importance of spacing.  **VHSND:** Provides counselling on spacing and limiting methods; where to access services; beneficiary incentives to women in reproductive age group specially newly married. ASHAs receive incentives under NHM. |
| **IV. Infection prevention** | | | | | | | | |
| Deworming prophylaxis | Yes (partially)  **Operational Guide for Implementing the National Maternal Infant and Young Child Nutrition (2019-2023):** Recommends taking deworming tablets every 6 months according to national protocols for pre-pregnant women. | No | Yes  **Bangladesh National Strategy for Maternal Health, 2019-30**  **National Strategy for Adolescent Health 2017-2030**  **National Plan of Action for Adolescent Health Strategy 2017-2030**  **National Strategy on Prevention and Control of Micronutrient Deficiencies 2015-24​**  All mention the provision of Iron and folate  supplementation  and de-worming. | No | Yes  **Bhutan Every Newborn Action Plan, 2016-2023:** Covers deworming for adolescent girls.  **Youth Friendly Health Services Guide and Implementation Standards​, 2008:** Covers treatment for worm infection | Yes (partially)  **Youth Friendly Health Services:** Covers deworming for adolescents. | Yes  **NDD Operational Guidelines, 2015:** Covers biannual dose of 400 mg albendazole (1 tablet) to both school-going and out-of-school adolescent girls ages 10-19 years  **Mass Drug Administration Guidelines, 2018:** a A combination of DEC (6 mg/kg body weight) + Albendazole (400 mg) in endemic areas free from onchocerciasis prevalence.  **AMB Operational Guidelines, 2018:** Newlywed/married women aged 20–24 years who are not pregnant or non-lactating will be provided biannual deworming during NDD.  **RKSK Implementation Guidelines, 2015 ​** : Covers biannual dose of 400 mg albendazole (1 tablet) to both school-going and out-of-school adolescent girls ages 10-19 years. | Yes (partially)  **NDD, RKSK** |
| Provision of bed nets in malaria endemic areas | Yes  **National Malaria Strategic Plan, 2018--2022** | Yes  **Basic Package of Health Services**  **Sehatmandi Project (UNDP, WHO and Global Fund)** | Yes  **National Strategic Plan for Malaria Elimination in Bangladesh, 2021-2025** | Yes  **National Malaria Elimination Programme** | Yes  **National Malaria Strategic Plan (NMSP) of Bhutan, 2020 – 2025​** | Yes  **National Malaria Eradication Program, 1964 (renamed in 2003 as National Malaria Control Programme)​** | Yes  **National Strategic Plan for Malaria Elimination, 2016-2030 ​** | Yes  **Malaria Elimination Program ​** |
| **V. Treatment and care for women at-risk^1^** | | | | | | | | |
| Social protection interventions (cash, food voucher, food ration) | No | No | No | No | No | No | No | No |
| Balanced energy and protein supplements in undernourished populations | Yes  **National Preconception Care​ Guideline, 2022**: Covers supplementing energy- and nutrient-dense food. | No | No | No | No | No | Yes  **INAP, 2014​:** Covers balanced energy protein supplementation at community-level under the preconception and antenatal package. | No |
| Contextualised dietary modification linked counselling tailored to nutrition assessment (under- or overweight/anaemia/ diabetes) | Yes  **National Preconception Care ​Guideline, 2022**: Mentions management of diabetes, including counselling people with diabetes mellitus. | No | No | No | Yes  **Bhutan Every Newborn Action Plan, 2016-2023:** Covers focused preconception counselling on a range of issues including obesity. | Yes  Universal delivery through all health facilities at districts level​. | Yes (partially)  **RKSK Operational Framework, 2014:** Counselling at AFHCs and management of low BMI, as per protocol, and referrals. | Yes (partially)  **RKSK:** Counselling at AFHCs and management of low BMI, as per protocol, and referrals. |
| Medical Nutrition Therapy, based on nutrition risk classification (underweight/ overweight/ anaemia/ diabetes)​ | No | No | No | No | Yes | Yes  Universal delivery through all health facilities at districts level​. | No | No |
| Anaemia with daily IFA supplementation for a period of 3 months | Yes (partially)  **National Micronutrient Guidelines**  **Operational Guide for Implementing the National Maternal, Infant and Young Child Nutrition (2019-2023):** Recommends universal IFA supplementation for adolescent girls aged 10-19 years and WRA with prevalence of severe anaemia ≥40%. | Yes (partially)  **Basic Package of Health Services (BPHS)**  **Essential Package of Hospital Services (EPHS)**  **​**  Both mention treatment of anaemia. | Yes  **National Guidelines: Prevention and Treatment of Iron Deficiency Anaemia, 2001;** National Strategy for​ Anaemia Prevention and Control in Bangladesh, 2007 | No | Yes  **Bhutan Every Newborn Action Plan, 2016-2023:** specifies treatment for anaemia  **Youth Friendly Health Services Guide and Implementation Standards, 2008:** Mentions iron therapy treatment to non-pregnant adolescents. | Yes  Universal through all health facilities at districts level.  **Youth Friendly Health Services ​** | Yes (partially)  **AMB Operational Guidelines, 2018**: Covers referral for severe and moderate cases from AHD to AFHCs. Through AFHCs, adolescents are managed with two IFA tablets (each with 60 mg elemental iron and 500 mcg folic acid), once daily, for 3 months, orally after meals for mild and moderate anaemia through AFHCs  **RKSK operational framework, 2014**​ | Yes (partially)  **AMB, RKSK** |
| Diabetes mellitus | Yes  **National Strategy for Prevention and Control of Non-Communicable  Diseases 2015-2020:**  Considers integration of NCDs such as prevention and control of cardiovascular diseases, diabetes, and chronic pulmonary diseases in the BPHS and EPHS.  **National Preconception Care ​Guideline, 2022**: Covers management of diabetes, including counselling people with diabetes mellitus. | Yes (partially)  **Basic Package of Health Services​**  **Essential Package of Hospital Services (EPHS)**  Both include treatment of diabetes. | Yes  **National Guideline on Diabetes Mellitus, July 2023 (First Edition)** | No  Universal programmes delivered through Upazila Health Complexes with no focus on preconception women​ | Yes  **Bhutan Every Newborn Action Plan, 2016-2023:​**Covers diagnosis and treatment of hypertension, diabetes, and hypothyroidism. It also includes focused preconception counselling on diabetes mellitus. | Yes (partially)  **Service with Care and Compassion Initiatives based on WHO package of essential NCDs (PEN)​** | Yes (partially)  **RKSK Operational Framework, 2014;**  **Prevention, Screening, and Control of Non-Communicable Diseases (HTN, Diabetes, and Common Cancers), Operational Guidelines, 2016:**  Recommends people with random blood sugar of 140mg/dl and more for referral to a medical officer at the nearest facility, for confirmation and initiation of treatment.  **Ayushman Bharat Health and Wellness Centres, Operational Guidelines, 2018​:**  Includes, diabetes, hypertension, mental health management for above 30 years. | Yes (partially)  **RKSK, National Programme for Prevention and Control of Non-Communicable Diseases:** Covers  screening and management of diabetes, hypertension, and mental health. |
| Depression and other psychosocial problems | Yes  **Mental Health Strategy 2019-2023**  **National Preconception Care Guideline, 2022​:** Covers counselling, treatment and management of depression in women planning pregnancy and other women of childbearing age. | Yes  **Basic Package of Health Services**  **Essential Package of Hospital Services (EPHS)**  Both mention treatment of mental health problems. | Yes  **Mental Health Act 2018**  **Mental Health Policy 2019**  **National Mental Health Strategic Plan 2020- 2030**  **National Strategy for Adolescent Health 2017-2030**  In 2018 Bangladesh Parliament approved a new Mental Health Act. A new Mental Health Policy, approved by the Ministry of Health in 2019, reflected a shift from a medical to a psychosocial treatment model with emphasis on decentralization and community-based services and support for persons living with mental illness.  **National Strategy for Adolescent Health 2017-2030:**  Promotes school and facility level interventions which include counselling and management of mental health disorders through linkage with the national mental health programme. | No  Universal programmes delivered through Upazila Health Complexes with no focus on preconception women​ | Yes  **Bhutan Every Newborn Action Plan, 2016-2023:** Covers mental health program as one of the interventions.  **Youth Friendly Health Services Guide and Implementation Standards​, 2008:** | Yes  Universal through all health facilities at districts level  **Youth Friendly Health Services:** Covers adolescents aged 10-24 years. Interventions include recognition of MH problems in the community and reporting and referral, diagnosis and treatment at the facility level, awareness raising on mental health at the community level. | Yes  **RKSK Operational Framework, 2014:** Mentions referral for adolescents with chronic/severe mental health issues to Adolescent Friendly Health Clinics (AFHCs) for regular counselling  **Guidelines for implementing district level activities under NMHP, 2015**: Includes screening and basic management (counselling and referral) of mental health ailments (Psychosis, Depression, Neurosis, Dementia, Mental Retardation, Autism, Epilepsy and Substance Abuse related disorders).  **Ayushman Bharat, Health and Wellness Centres, Operational Guidelines​, 2018** | Yes  **RKSK**  **Ayushman Bharat:** Screening and basic management of mental health ailments (Psychosis, Depression, Neurosis, Dementia, Mental Retardation, Autism, Epilepsy and Substance Abuse related disorders) at the community level and health and wellness centres. |
| STI/RTI | Yes  **National Reproductive, Maternal, Newborn, Child, and Adolescent Health Strategy 2024-2028**  **National Preconception Care​**  **Guideline, 2022**: Mentions increasing access to treatment for STI | Yes (partially)  **Basic Package of Health Services**  **Essential Package of Hospital Services (EPHS):** Mentions treatment of STIs. | Yes  **Bangladesh National Strategy for Maternal Health, 2019-30**  **National Strategy for Adolescent Health 2017-2030:** Mentions about information provision to prevent STIs, HIV to all pre-pregnant women. | No  Universal programmes delivered through Upazila Health Complexes with no focus on preconception women​ | Yes  **Bhutan Every Newborn Action Plan, 2016-2023:** Specifies prevention and treatment of STIs/HIV.  **Youth Friendly Health Services Guide and Implementation Standards** | Yes  Universal through all health facilities at districts level  **Youth Friendly Health Services:** Covers adolescents 10-24 years through community and BHU2/outreach clinics. | Yes  **RKSK Operational Framework, 2014** - Referral to AFHCs for adolescents with symptoms of RTI/STI from AHD and management at AFHCs  **National Guidelines for VHSND, 2019:** Referral to ICTC while ensuring confidentiality and counselling on prevention of RTI and STIs, including HIV/AIDS. | Yes  **RKSK:** Referral to AFHCs for adolescents with symptoms of RTI/STI from AHD and management at AFHCs  **VHSND**: Referral to ICTC while ensuring confidentiality |

^1^Underweight (BMI <18.5 kg/m2); Overweight/obese (BMI >=25 kg/m2); Short stature (height <145 cm); Anaemia (Hb <=12 g/dl); Diabetic (blood sugar level >140 mg/dl ); Having mental health concerns; Having STI/RTIs

| **Interventions** | **Maldives** | | **Nepal** | | **Pakistan** | | **Sri Lanka** | |
| --- | --- | --- | --- | --- | --- | --- | --- | --- |
|  | **Policy** | **Program** | **Policy** | **Program** | **Policy** | **Program** | **Policy** | **Program** |
| **I. Health and Nutrition Assessment** | | | | | | | | |
| Anthropometry (height, weight, or waist to hip ratio) | Yes  **National Guideline on Antenatal and Postnatal Care in the Maldives, 2022:**  Recommends measurement of weight and BMI for women before pregnancy during preconception counselling or contraceptive consultations at Public Health Unit (PHU) and Reproductive Health Centres (RHC). | Yes (partially)  **Adolescent and Youth Friendly Health Services:**  Assesses BMI of adolescents in adolescent friendly clinics. | Yes  **ANC to PNC Continuum of Care Guidelines, 2022:** Suggests height and weight measurement to assess nutritional status of prepregnant women.  **National Adolescent Health Development Strategy-2000 (revised in 2018):**  Suggests health screening and counselling services through schools, clubs, and other community-based organizations.  **National Adolescent Sexual and Reproductive Health Strategy, 2007:**  Suggests anthropometric assessments in schools. | No | Yes  **Pakistan Maternal Nutrition Strategy, 2022-27:** Suggests designing and implementing preconception care under maternal nutrition interventions and one of the activities outlined is, monitoring nutritional status of women before pregnancy.  **Pakistan Adolescent Nutrition Strategy and Operational Plan, 2020-25:**  Suggests holding frequent screening camps for adolescents in schools, hospitals and communities to assess anthropometry and anaemia status. | No | Yes  **National Maternal and Child Health Policy of Sri Lanka 2012**  **Basic Maternal Care: A Guide for Health Care Providers 2023:**  Mentions checking Body Mass Index (BMI) and taking necessary interventions according to the BMI. | Yes  **Service Package for Newly Married Couples** |
| Anaemia (Blood test such as haemoglobin) | Yes  **National Guideline on Antenatal and Postnatal Care in the Maldives, 2022:**  Suggests screening for anaemia as one of the preconception care interventions. | No | Yes  **ANC to PNC Continuum of Care Guidelines, 2022:**  Suggests anaemia screening for women during the preconception period**.**  **National Adolescent Health Development Strategy-2000 (revised in 2018):**  Suggests health screening and counselling services through schools, clubs, and other community-based organizations.  **National Adolescent Sexual and Reproductive Health Strategy, 2007:**  Recommends selected facilities to provide screening for anaemia through haemoglobin estimation. | No | Yes  **Pakistan Maternal Nutrition**  **Strategy, 2022-27:**  Suggests designing and implementing preconception care under maternal nutrition interventions and one of the activities outlined is, screening for anaemia and diabetes, and the management of both conditions.  **Pakistan Adolescent Nutrition Strategy and Operational Plan, 2020-25:**  Suggests holding frequent screening camps for adolescents in schools, hospitals and communities to assess anthropometry and anaemia status. | No | Yes  **National Maternal and Child Health Policy of Sri Lanka, 2012**  **Basic Maternal Care; A Guide for Health Care Providers 2023:**  Recommends haemoglobin estimations and if anaemic, taking necessary actions. | Yes  **Service Package for Newly Married Couples** |
| Diabetes mellitus using oral glucose tolerance test | Yes  **National Guideline on Antenatal and Postnatal Care in the Maldives, 2022:**  Suggests screening for diabetes as preconception care in women and couples.  **Primary Health Care Model for Universal Coverage, 2022**  **(Essential package of services for PHC):**  Suggests screening and treatment for risk of CVD/diabetes in all population. | Yes (partially)  **Primary Health Care Model in Faafu Atoll (pilot), 2022:** Screening for risk of diabetes in all population.  **Adolescent and Youth Friendly**  **Health Services:**  Provides random blood sugar test for adolescent and youth aged 10-24 years through monthly mobile visits by specialist doctors from a higher centre (regional/atoll) at health post. | Yes  **Multisectoral Action Plan for the**  **Prevention and Control of Non-Communicable Diseases (2014-2020)​:** Adapts package of WHO Package of essential non- communicable disease interventions to screen, diagnose, treat and refer for NCDs including diabetes at VDC (village development committees), PHC and hospital levels.  **ANC to PNC Continuum of Care Guidelines, 2022:**  Suggests diabetes screening for women during the preconception period**.** | Yes  **Package of Essential Non-communicable Diseases (PEN)​:**  Detects NCDs including diabetes, provides counselling, referral, follow up of low-risk patients, refill drugs, health promotion at health post.  Confirms diagnosis of diabetes, manages, and follows up high risk patients at PHCs, and refers complicated cases to districts hospital. | Yes  **Pakistan Maternal Nutrition Strategy, 2022-27:** Suggests designing and implementing preconception care under maternal nutrition interventions and one of the activities outlined is, screening for anaemia and diabetes, and the management of both conditions. | No | Yes  **Basic Maternal Care; A Guide for Health Care Providers 2023**  **Guidelines for the delivery of "Service Package for Newly Married Couples, 2018":**  Recommends checking blood sugar level in both partners. | Yes  **Service Package for Newly Married Couples** |
| Sexually transmitted infections (STIs) or reproductive tract infections (RTIs) | Yes  **National Guideline on Antenatal and Postnatal Care in the Maldives, 2022:**  Suggests screening for STIs as preconception care in women and couples.  **National Standards for Family Planning Services, 2019:**  Recommends STI/RTI assessments during initial family planning visits using history taking and/or clinical examination prior to initiating family planning in all women.  **Maldives National Reproductive,**  **Maternal, Newborn, Child and Adolescent Health (RMNCAH) Strategy and Action Plan (2020-2025):**  Aims to increase lab capacity for testing and treatment in tertiary, regional, and atoll hospitals and capacity building of service providers for STI screening and testing. | Yes  **Adolescent and Youth Friendly**  **Health Services:**  Assesses STI/RTIs (by CHW /nurse/DR) at all levels and follows national guideline for management.  **Routine family planning visits:**  Assesses STI/RTIs during routine family planning visits using history taking and/or clinical examination prior to initiating family planning in all women. | Yes  **National Guidelines on Management of Sexually Transmitted Infections, 2022:**  Provides steps for clinical examination of STI in both men and women at health facilities, along with treatment, follow-up and referral to higher level of health facilities. | Yes  **National HIV/AIDS and STI Control Program:** Provides  HIV and STI counselling and testing up to the health post level and through standalone HIV Testing Centre sites run by NGOs. | Yes  **The National Guidelines for the Management of Sexually Transmitted Infections, 2007:**  Guides history taking and examination to diagnose the infection. | Yes (partially)  **Essential Package of Health Services (EPHS):**  Provides testing for STIs for all in contact with health system in high prevalence settings. | Yes  **Basic Maternal Care; A Guide for Health Care Providers 2023**  **Guidelines for the delivery of "Service Package for Newly Married Couples, 2018":**  Recommends screening of depression and other psychological concerns by the public health staff. | Yes  **Service Package for Newly Married Couples** |
| Psychosocial problems | Yes  **National Guideline on Antenatal and Postnatal Care in the Maldives, 2022:**  Suggests assessment of psychosocial problems under preconception care interventions.  **National Mental Health Policy 2015-25:**  Aims to increase awareness and strengthen mental health services (screening, referral and treatment) through a community- based approach.  **National Mental Health Strategic Plan 2016-21:**  Suggests sensitizing and training the mental health professionals and non-mental health groups (e.g., primary health care staffs etc.) for early identification, support and for facilitating referrals, follow up in the community. And supporting primary care services and assisting with the provision of community-based mental health care.  **Maldives National Reproductive,**  **Maternal, Newborn, Child and Adolescent Health (RMNCAH) Strategy and Action Plan (2020-2025):**  Suggests providing mental health services such as identification, treatment, and referral along with counselling in accordance with the national plan to adolescents and youth at regional and Atoll levels. And increasing frequency and coverage of regular comprehensive health screening (nutrition, mental health) of school, college and university students. | Yes (partially)  **Adolescent and Youth Friendly Health Services:**  Provides mental health assessment and history taking at the primary level and referral to higher levels for diagnosis. | Yes  **National Mental Health Policy 1996 (revised in 2017):**  Advocates the integration of mental health services into primary health care system to address the mental health and psychosocial needs of the people.  **The National Mental Health Strategy and Action Plan (2020):** Recommends integration of mental health in primary health and strengthen mental health services at the secondary health care level.  **Multi-sectoral Action Plan for the Prevention and Control of Non-Communicable Diseases (2014-2020):​** Suggests improving competency for case identification and initiating referral at primary care level.  **National Health Policy, 2019:**  Mentions ensuring access to mental health and psychosocial services for all through PHC by capacity building and trainings. | Yes  Integration of mental health services and psychosocial counselling in primary healthcare (Community-based mental health and psychosocial Integration)​  **Community Mental Health Care Program, 2017 (adapted the mhGAP tools):**  Detects individuals with mental health and psychosocial problems through screening and assessment at health facilities. | Yes (partially)  **Pakistan Adolescent Nutrition Strategy and Operational Plan, 2020-25:**  Plans to assess behavioural profiles, dietary patterns, cost of the diet and major influencers of adolescents in the context of their social and psychosocial development. | No | Yes  **Basic Maternal Care; A Guide for Health Care Providers 2023**  **Guidelines for the delivery of "Service Package for Newly Married Couples, 2018"** | Yes  **Service Package for Newly Married Couples** |
| **II. Provision of essential micronutrients** | | | | | | | | |
| Folic acid supplements, if planning pregnancy | No | No | Yes  **National Medical Standard for Maternal and Newborn Care, 2022:**  Recommends daily folic acid to prevent neural tube defects. | Yes  Folic acid supplementation programme for newly married women. | Yes  **Pakistan Maternal Nutrition Strategy, 2022-27:**  Suggests designing and implementing preconception care under maternal nutrition interventions and one of the activities outlined is, supplementation with either folic acid or iron and folic acid. | No | Yes  **National Maternal and Child Health Policy of Sri Lanka, 2012**  **National Strategic Plan Maternal and Newborn Health (2017 -2025)**  **Basic Maternal Care; A Guide for Health Care Providers 2023**  **Guidelines for the delivery of "Service Package for Newly Married Couples", 2018**  All recommend daily folic acid supplementation for women planning pregnancy. | Yes  **Service Package for Newly Married Couples** |
| Supplementation with iron containing supplements (Iron Folic Acid or IFA) ​ | Yes  **Maldives National Reproductive,**  **Maternal, Newborn, Child and Adolescent Health (RMNCAH) Strategy and Action Plan (2020-2025):**  Recommends intermittent IFA supplementation to menstruating girls and women where anaemia is a public health problem.  **National Guideline on Antenatal and Postnatal Care in the Maldives, 2022:**  Suggests IFA under the preconception care interventions.  **Primary Health Care Model for Universal Coverage, 2022**  **(Essential package of services for PHC):**  Recommends intermittent IFA supplementation for menstruating girls and women where anaemia is a public health problem. | Yes (partially)  **Adolescent and Youth Friendly Health Services:**  Provides micronutrient supplements at primary health centres to adolescent and youth aged 10-24 years. | Yes  **National Strategy for the Control of Anaemia Among Women and Children in Nepal, 2007: Suggests** reviewing the possibility of extending the distribution of IFA to the groups at risk such as small children, adolescents and women of reproductive age.  **National Adolescent Health and Developmental Strategy, 2000 (revised in 2018):**  Suggests IFA to adolescent girls aged 10-19 years including married girls.  **ANC to PNC Continuum of Care Guidelines:**  Suggests IFA supplementation to women during the preconception period. | Yes (partially)  **Weekly Iron and Folic Acid Supplementation**  **Programme, 2016:** Provides IFA to out of school adolescent girls between 10 to 19 years of age through facilities. | Yes  **Pakistan Maternal Nutrition Strategy, 2022-27:**  Suggests designing and implementing preconception care under maternal nutrition interventions and one of the activities outlined is, supplementation with either folic acid or iron and folic acid. | Yes  Provision of standard IFA if planning pregnancy. | No | No |
| Large-scale food fortification of staple foods | Yes  **Maldives National Reproductive, Maternal, Newborn, Child and Adolescent Health (RMNCAH) Strategy and Action Plan (2020-2025):**  Suggests fortifying foods such as flour and/or rice with iron and folic acid for prevention of iron deficiency anaemia among WRA, children and adolescents and subsidising the cost of these fortified foods to incentivise use by the general public. | No | Yes  **Multisector Nutrition Plan (2018-2022)**  **Iodized Salt Act (1998):** Outlines the control, licencing, standards and oversight for importation of iodized salt, and in essence has made iodization of all salt intended for human consumption mandatory.  **National Nutrition Policy and Strategy, 2004** | Yes  **Universal Salt Iodization (USI) Program** | Yes  **Pakistan Multi-sectoral Nutrition Strategy 2018-2025:** Recommends fortifying staple foods including cooking oils, wheat flour and iodized salt.  **Pakistan Adolescent Nutrition Strategy and Operational Plan, 2020-25:**  Includes provision of additional micronutrients through fortification of staple foods such as wheat (with iron, folic acid, zinc, vitamin B12), oil (with vitamins A and D) and salt with iodine.  **Pakistan National Food Fortification Strategy, 2017**  **Food fortification bill for KP, Baluchistan:** Fortifies wheat with 4 multi-micronutrients, including salt, wheat and oil. | Yes  **Universal Salt Iodization Programme, 1994**  **Pure food rule mandatory fortification** **of oil/ghee** with vitamin D and A. | Yes  **National Nutrition Policy, 2021-30:**  Directs to enhance the nutrient content of staple/ essential food by fortification/ bio-fortification as appropriate.  **National Strategy for Prevention and Control of Micronutrient Deficiencies in Sri Lanka 2017-2022:**  Suggests developing and implementing policy on food fortification (such as flour, rice, salt) with iron and appropriate micronutrients; strengthening the salt iodization programmes**;** promoting fortification of staple foods or commonly consumed condiments/spice to increase iron and appropriate micronutrient intake of the population.  **Regulations under the Food Act (1980), 2005:**  Bans manufacture, import, store for sale, sell, offer for sale, expose or keep for sale, transport or distribute any type of edible common salt other than iodized or iodated common salt for human consumption, including salt used as an ingredient of food and for food manufacture.  **Food (Refined Wheat Flour Fortification) Regulations, 2022: Regulates the folic acid and iron fortification requirements of refined wheat flour for retail sale for domestic consumption and bread manufacturing.** | Yes (partially)  **USI, 1995**: Mandatory salt iodization at a level of 25 ppm.  **Food (Iodisation of Salt) Regulations under the Food Act (1980), 2005** |
| **III. Counselling on healthy eating and family planning choices and services** | | | | | | | | |
| Counselling on healthy eating and physical activity | Yes  **Maldives National Reproductive, Maternal, Newborn, Child and Adolescent Health (RMNCAH) Strategy and Action Plan (2020-2025):**  Suggests strengthening counselling on dietary intake and healthy lifestyle during ANC, PNC and family planning visits and increase community and family awareness on importance of women’s nutritious and safe diets, negative effects of malnutrition, IFA supplementation, and fortified food. | Yes  **Premarital session:** Conducted with newly married couples at the family court, passed down by magistrate or health workers on different topics such as nutrition, health, etc.  **Adolescent and Youth Friendly Health Services:**  Information provision on balance diet, exercise, and micronutrient deficiency. | Yes  **Multisector Nutrition Plan (2018-2022)**  **National Adolescent Sexual and Reproductive Health Strategy, 2007:**  Suggests service providers to provide information on balanced diet and consumption of green leafy vegetables, and other iron rich foods.  **ANC to PNC Continuum of Care Guidelines, 2022:**  Suggests counselling on nutrition and physical activity to women during preconception period. | Yes  **NCD Prevention Program (WHO and government)**:  Counselling on healthy eating and promotion of physical activity provided by health worker to women who attend health facility. | Yes  **Pakistan Maternal Nutrition Strategy, 2022-27:**  Suggests designing and implementing preconception care under maternal nutrition interventions and one of the activities outlined is counselling on healthy, diverse diets, and physical activity. | No | Yes  **National Nutrition Policy, 2021-30:**  Directs provision of pre-pregnancy care for the couple before planning their first child or to plan subsequent pregnancies and to enter pregnancy with optimum nutrition in a supportive environment.  **National Maternal and Child Health Policy of Sri Lanka, 2012**  **The National Policy and Strategic Framework for Prevention and Control of Chronic Non-Communicable Diseases 2023-2033**  **National Strategic Plan for Maternal and Newborn Health (2017 -2025)**  **Basic Maternal Care, A Guide for Health Care Providers 2023:**  Recommends including nutrition related topics in preconception care sessions such as appropriate diet for underweight and overweight/ and obesity for newly married couples. | Yes  **Service Package for Newly Married Couples, 2018** |
| Reduce caffeine/ alcohol/ smoke / toxin exposure | Yes  **National Child Health Strategy – Every Newborn Action Plan (ENAP) Maldives, 2016-2020:**  Recommends prevention of substance abuse during preconception period.  **National Guideline on Antenatal and Postnatal Care in the Maldives, 2022:** Suggests screening women and men in preconception period and advising about harm of second-hand smoke and harmful effects on pregnant women and unborn children. | Yes (partially)  **Adolescent and Youth Friendly Health Services:**  Assesses substance abuse, provides psychosocial counselling and referral at all levels. | Yes (partially)  **Multi-sectoral Action Plan for Prevention and Control of NCDs (2014-2020)​**  **Tobacco Products (Control and Regulatory) Act, 2011**  **National Policy on Regulation and Control of Alcohol, 2017** | Yes (partially)  **National Tobacco Control Program** | No | No | Yes  **Guidelines for the delivery of "Service Package for Newly Married Couples, 2018:**  Recommends including importance of avoiding harmful practices such as smoking, alcohol consumption and helps couples to change behaviour during preconception care sessions. | Yes  **Service Package for Newly Married Couples, 2018:**  Includes the following in the preconception care sessions- impact of smoking, drinking and toxin exposure for both mother and baby, avoiding harmful practices such as smoking, alcohol consumption, avoiding tea and coffee just before and after meals  (as mentioned in the booklet for newly married couples and handbook to guide health staff on healthcare). |
| Psychosocial counselling | Yes  **National Guideline on Antenatal and Postnatal Care in the Maldives, 2022:**  Suggests providing educational and psychosocial counselling before and during pregnancy.  **National Mental Health Policy 2015-2025:**  Aims to increase awareness about mental health in the community through campaigns targeted to different populations using different media and age appropriate strategies.  **National Mental Health Strategic Plan 2016-21:**  Aims to increase awareness about mental health through social media messages and campaigns; review of pre-marital package to include emotional well-being of families, communication skills, stress management, coping, conflict resolution, anger management skills,  fatherhood and motherhood programs, etc.  **Maldives National Reproductive,**  **Maternal, Newborn, Child and Adolescent Health (RMNCAH) Strategy and Action Plan (2020-2025:**  Suggests increasing awareness of adolescents and youth on mental health issues such as depression, anxiety, stress management, positive behaviours, safe environment, problem solving and coping skills etc. and promoting awareness raising campaigns to reduce stigma and accessing mental health services. | Yes (partially)  **Adolescent and Youth Friendly Health Services:**  Provides preventive care and awareness programmes at all levels of health facility. | Yes  **National Mental Health Policy 1996 (revised in 2017):**  Advocates the integration of mental health services into primary health care system to address the mental health and psychosocial needs of the people.  **The National Mental Health Strategy and Action Plan (2020):**  Recommends integration of mental health in primary health and strengthens mental health services at the secondary health care level. | Yes  **Community Mental Health Care Package, 2017:** Provides awareness within the community about mental health, psychosocial problems, and available services by female community health volunteers, mother’s groups, teachers, local leaders, traditional faith healers, community-based organizations. | No | No | Yes  **Basic Maternal Care; A Guide for**  **Health Care Providers 2023**  **Guidelines for the delivery of "Service Package for Newly Married Couples, 2018":**  Recommends awareness generation sessions on the importance of mutual exchange or ideas, controlling arrogance and mental stress and help couples understand each other. | Yes  **Service Package for Newly Married Couples** |
| Family planning choices for delaying age at first pregnancy (>=20 years) / increase inter-birth interval (at least 24 months) | Yes  **National Child Health Strategy – Every Newborn Action Plan (ENAP) Maldives, 2016-2020:**  Suggests revising the national family planning standards and guidelines with emphasis on birth spacing and develop communication strategies to prevent early marriages and pregnancy among adolescents.  **National Standards for Family Planning Services, 2019:**  Recommends range of family planning methods available in the country along with counselling services to ensure clients make an informed choice.  **National Reproductive Health Strategy 2014-2018:**  Suggests increasing awareness among couples, brides-bridegrooms to be and adolescents on the importance of using contraceptives to prevent unwanted pregnancy and ensure availability of contraceptives and improve contraceptive choice in all service delivery points. | Yes  **National Family Planning Programme, 1986:**  Provides a range of family planning methods available in the country along with counselling services to ensure clients make an informed choice. | Yes  **Multi-Sector Nutrition Plan, 2018-2022:** One of the interventions under enhancing nutritional status of WRA including adolescents is, raising awareness and services for healthy timing and spacing of pregnancy and increasing access to and utilization of family planning tools  **National Adolescent Health and Developmental Strategy, 2000:**  Includes family planning counselling to married adolescent girls aged 10-19 years.  **National Strategy for Family Planning Services, 2068**    **Nepal Health Sector Strategy 2015–2020:**  Specifies all WRA should receive various family planning services free of cost through government health facilities.  **National Adolescent Sexual and Reproductive Health Strategy, 2007**: Recommends enrolling newly married adolescent couples and provides counselling and spacing methods during routine sub centre clinics.  **ANC to PNC Continuum of Care Guidelines, 2022:** Mentions counselling and provision of family planning methods for birth spacing and to delay pregnancy. | Yes  **Family Planning Programme:**  Provides free counselling and family planning commodities at all public institutions  **National Adolescent Sexual and Reproductive Health Program, 2011:**  Enrols newly married couples and provides spacing methods at sub centres. | Yes  **National Integrated Reproductive, Maternal, Newborn, Child, Adolescent Health & Nutrition Strategy (2016-2020)**  **Pakistan Multi-Sectoral Nutrition Strategy, 2018-25** | Yes  **National Program for Family Planning**  **Primary Health Care, EPHS:**  Provides condoms and hormonal contraceptives including IUDs and emergency contraceptives | Yes  **National Maternal and Child Health Policy of Sri Lanka, 2012:** Directs all couples to have a desired number of children with optimal spacing whilst preventing unintended pregnancies through a range of strategies.  **National Strategic Plan for Maternal and Newborn Health (2017 - 2025):** Strengthens pre-pregnancy, inter-pregnancy and maternal care programmes to empower couples for appropriate use of contraception to plan pregnancies.  **Population and Reproductive Health Policy, 1998** | Yes  **Service Package for Newly Married Couples, 2018:** Provides appropriate family planning services including counselling to delay the 1st child in newly married couples and spacing the subsequent births.  **National Family Planning Program** |
| **IV. Infection prevention** | | | | | | | | |
| Deworming prophylaxis | No | No | Yes (partially)  **National School Health and Nutrition Strategy 2006** | Yes (partially)  **School Health and Nutrition Program**: Provision of deworming tablets to married adolescent girls who attend schools.  **Female Community Health Volunteer Program:** Distribution of deworming to WRA in rural areas in some provinces. | Yes (partially)  **Pakistan Adolescent Nutrition Strategy and Operational Plan 2020-2025:** Suggests providing preventive deworming for adolescents through health facilities and schools. | No | Policy not required | Programme not required |
| Provision of bed nets in malaria endemic areas | Policy not required | Programme not required | Yes  **Nepal Malaria Strategic Plan, 2014-25:**  Suggests mass distribution of Long-Lasting Insecticide Treated Nets (LLINs) to all population groups in high-risk areas. | Yes  **National Malaria Control Program:**  Mass distribution of Long-Lasting Insecticide Treated Nets (LLINs) to all population groups in high-risk areas. | Yes  **National Strategic Plan for Malaria Elimination,  2021-35**  Suggest mass distribution of Long-Lasting Insecticide Treated Nets (LLINs)  to all populations in high-risk areas. | Yes  **National Malaria Control Programme, 1950 / Malaria Eradication Programme, 1961:**  Mass distribution of Long-Lasting Insecticide Treated Nets (LLINs to entire populations in high-risk areas. | Policy not required | Programme not required |
| **V. Treatment and care for women at-risk^1^** | | | | | | | | |
| Social protection interventions (cash, food voucher, food ration) | No | No | No | No | No | No | No | No |
| Balanced energy and protein supplements in women of reproductive age in undernourished populations | No | No | Yes  **ANC to PNC Continuum of Care Guidelines:**  Suggest BEP supplementation to women during the preconception period. | No | Yes  **Pakistan Maternal Nutrition Strategy, 2022-27:** Suggests designing and implementing preconception care under maternal nutrition interventions and one of the activities outlined is supplementing diets with energy and nutrient dense foods for malnourished women. | No | No | No |
| Contextualised dietary modification linked counselling tailored to nutrition assessment (under- or overweight/anaemia/ diabetes) | Yes  **National Guideline on** **Antenatal and Postnatal Care in the Maldives, 2022:** Recommends personalized approach to weight concern and lifestyle for overweight/obese WRA during pre and inter-pregnancy periods to normalize weight and referral to dietitian. | Yes (partially)  **Primary Health Care model in Faafu Atoll (pilot), 2022:** Includes a comprehensive set of services including screenings at population level for selected non-communicable diseases, lifestyle counselling services, identification and referral for common cancers and selected mental health conditions.  **Adolescent and**  **Youth Friendly**  **Health services:**  Provision of contextualized counselling based on the specific case (NCD, micronutrient deficiency etc). | No | No | Yes  **Pakistan Maternal Nutrition Strategy, 2022-27** | No | Yes  **Basic Maternal Care; A Guide for Health Care Providers 2023:** Provision of counselling based on nutritional assessment. | Yes  **Service Package for Newly Married Couples** |
| Medical Nutrition Therapy, based on nutrition risk classification (underweight/ overweight/ anaemia/ diabetes)​ | No | No | No | No | No | No | Yes | Yes |
| Anaemia with daily IFA supplementation for a period of 3 months | No | No | No | No | Yes  **Pakistan Maternal Nutrition Strategy, 2022-27:** Suggests designing and implementing preconception care under maternal nutrition interventions and one of the activities outlined is screening for anaemia and diabetes, and the management of both conditions. | No | Yes  **Basic Maternal Care; A Guide for Health Care Providers 2023:** Provision of anaemia treatment. | Yes  **Service Package for Newly Married Couples** |
| Diabetes mellitus | Yes  **National Guideline on Antenatal and Postnatal Care in the Maldives, 2022:**Recommends preconception counselling for women of reproductive age with diabetes mellitus.  **Primary Health Care Model for Universal Coverage, 2022**  **(Essential package of services for PHC):** Suggests screening and treatment for risk of CVD/diabetes in all population. | Yes (partially)  **Primary Health Care Model in Faafu Atoll (pilot), 2022:** Includes a comprehensive set of services including screenings at population level for selected non-communicable diseases including diabetes, lifestyle counselling services, identification and referral for common cancers and selected mental health conditions. | Yes  **Multi-sectoral Action Plan for the Prevention and Control of Non-Communicable Diseases (2014-2020)​:** Adapts the WHO Package of Essential Non- Communicable Disease; includes interventions such as screening, diagnoses, treatment and referral for NCDs including diabetes at VDC (village development committees), PHC and hospital levels.  **ANC to PNC Continuum of Care Guidelines, 2022:**  Suggests management and counselling of women with diabetes during preconception period. | Yes  **Package of Essential Non-communicable Diseases (PEN):​**  Detects NCDs including diabetes, counselling, referral, follow up of low-risk patients, refill drugs, health promotion at health post.  Confirms diagnosis of diabetes, manage, follow up high risk patients at PHCs, refer complicated cases to districts hospital. | Yes  **Pakistan Maternal Nutrition Strategy, 2022-27:** Suggests designing and implementation of preconception care under maternal nutrition interventions and one of the activities outlined is, screening for anaemia and diabetes, and the management of both conditions. | No | Yes  **Guidelines for the delivery of "Service Package for Newly Married Couples, 2018"**: Provision for treatment of diabetes. | Yes  **Service Package for Newly Married Couples** |
| Depression and other psychosocial problems | Yes  **National Guideline on Antenatal and Postnatal Care in the Maldives, 2022:**  Recommends counselling, treatment, and management of depression in women planning pregnancy and other women of childbearing age.  **National Mental Health Policy 2015-25:** Aims to increase awareness and strengthen mental health services (screening, referral and treatment, counselling) through a community- based approach.  **Primary Health Care Model for Universal Coverage, 2022**  **(Essential package of services for PHC):** Provision of basic treatment for people with depression, anxiety and other psychosocial problems. | Yes (partially)  **Adolescent and Youth Friendly Health Services:**  Provision of referral and counselling by a trained counsellor at higher levels upon diagnosis.  **Primary Health Care model in Faafu Atoll (pilot), 2022:** Includes a comprehensive set of services including screenings at population level for selected non-communicable diseases, lifestyle counselling services, identification and referral for common cancers and selected mental health conditions. | Yes  **National Mental Health Policy 1996 (revised in 2017):**  Advocates for the integration of mental health services into the primary health care system to address mental health and psychosocial needs of the people.  **The National Mental Health Strategy and Action Plan (2020):** Recommends integration of mental health in primary health and strengthen mental health services at the secondary health care level.  **Multi-sectoral Action Plan for the Prevention and Control of Non-Communicable Diseases (2014-2020):​** Suggests improving competency for case identification and initiating referral at primary care level.  **National Health Policy 2019**: Mentions ensuring access to mental health and psychosocial services for all through PHC by capacity building and trainings. | Yes  Integration of mental health services and psychosocial counselling in primary healthcare.  **Community Mental Health Care Package, 2017**: Provision of treatment and psychosocial support, follow up and referral services at health facilities. | Yes (partially)  **Pakistan**  **Adolescent Nutrition Strategy and Operational Plan, 2020-25** | Yes (partially)  **Essential Package of Health Services:**  Management of depression and anxiety disorders with psychological and generic antidepressant therapy for all population. | Yes  **Guidelines for the delivery of "Service Package for Newly Married Couples, 2018":** Provision of prevention, identification and management of depression. | Yes  **Service Package for Newly Married Couples** |
| STI/RTI | Yes  **National Standards for Family Planning Services (2019):**  Recommends etiologic management (education and counselling) as well as treatment of women with symptomatic partners and arranges treatment of partners.    **Maldives National Reproductive,**  **Maternal, Newborn, Child and Adolescent Health (RMNCAH) Strategy and Action Plan (2020-2025:** Aims to increase lab capacity for testing and treatment in tertiary, regional, and atoll hospitals and capacity building of service providers to do STI screening and testing. | Yes  **Routine family planning visits:** Provision of treatment of women with symptomatic partners and for their partners.  **Adolescent and**  **Youth Friendly**  **Health Services:**  Provision of one on one counselling to at risk couples/ individuals, referral for diagnosis and treatment at higher facilities (managed as per the national guideline). | Yes  **National Guidelines on Management of Sexually Transmitted Infections, 2022**: Provision of clinical examination of STI in both men and women at health facilities, along with treatment, follow-up and referral to higher level of health facilities.  **National Adolescent Sexual and Reproductive Health Strategy, 2007:** Provision of treatment, follow up visits and referral for common STI/RTIs in both married and unmarried adolescents. | Yes  **National HIV/AIDS and STI Control Program:** Provision of counselling and testing for  HIV and STI up to the health post level and through standalone HTC sites run by NGOs. | Yes  **The National Guidelines for the Management of Sexually Transmitted Infections, 2007:** Guides syndromic management of STI/RTIs in health facilities according to WHO guidelines. | Yes (partially)  **Essential Package of Health Services:** Provision of syndromic management of common STI/RTIs according to WHO guidelines including partner’s treatment. | Yes  **Guidelines for the delivery of "Service Package for Newly Married Couples, 2018":** Provision of STI/RTI treatment | Yes  **Service Package for Newly Married Couples** |

^1^Underweight (BMI <18.5 kg/m2); Overweight/obese (BMI >=25 kg/m2); Short stature (height <145 cm); Anaemia (Hb <=12 g/dl); Diabetic (blood sugar level >140 mg/dl ); Having mental health concerns; Having STI/RTIs

**S3. Systems bottleneck classification criteria for each system building block**

| **System building block** | **Classification criteria for severity of bottleneck** | | | |
| --- | --- | --- | --- | --- |
|  | **Significant bottleneck** | **Moderate bottleneck** | **Mild bottleneck** | **No bottleneck** |
| 1. **Legislation and policies** |  |  |  |  |
| Are the policies/ legislations been translated into a program with operational guidelines (including financial guidelines, funded by government)?   - *Is there a legislation/ policy available to implement the intervention?* - *Has the legislation been translated into a program with operational guidelines (including financial guidelines, funded by government)?* | Only pronouncement has been made but no legislation (act) /Policy | Legislation/policy available, translated into programme with no operational framework/ guidelines and grossly inadequate financial guideline to support implementation | Legislation/policy available, translated into programme with an operational guideline in place but insufficient related to implementation | Legislation/policy available, translated into programme with an operational guideline in place with financial guidelines/costs to support implementation |
| 1. **Leadership, governance, and coordination** |  |  |  |  |
| ***If*** “yes” for Q1 then, is there leadership at national level to oversee targets and review the program with institutional architecture and administrative governance to support implementation, collaboration within and across sectors/ stakeholders/ advocates/ civil society?   - *Is there a national level/highest level leadership and coordinating mechanism which meets regularly overseas targets and comprehensive progress review of the [programme]?* - *Is there a national level/highest level leadership and coordinating mechanism which meets regularly overseas targets and comprehensive progress review of the [programme]?* - *Are there administrative governance processes established for collaboration/ consensus seeking/ stocktake for implementation of programme (within health sector, across sectors, across stakeholder groups)?* - *Is the institutional system/architecture in place to support programme implementation?* - *Are performance evaluation mechanisms in place and functional to establish accountability in the system?* - *Is there a presence of high-level advocates/civil society participation to ensure people's voices and accountability for duty bears?* | Leadership and coordinating mechanism not in place for implementation | Leadership and coordinating mechanism in place, but 2 or more challenges in implementation of established processes and systems (regarding coordination, collaboration/coalition, performance evaluation, advocacy) | Leadership and coordinating mechanism in place, but at least one challenge in implementation of established processes and systems (regarding coordination, collaboration/coalition, performance evaluation, advocacy) | Leadership and coordinating mechanism in place and this is being implemented (established processes and systems for coordination, collaboration/coalition, performance evaluation, advocacy) |
| 1. **Budget and financing** |  |  |  |  |
| Are the programs/interventions budgeted? If “yes” then, allocated and disbursed efficiently; tracked; increased budgetary provisions over time?   - *Is there a budget allocation line for the programme which includes all interventions?* - *Is the allocative efficiency sufficient and includes all aspects of programme systems to implement the interventions?* - *Is there disbursement efficiency/ adequate to cover disparities?* - *Are there process instituted to support tracking allocation, disbursement and allocative efficiency and are the budgetary provisions increasing over time?* | No budget allocation line for interventions available | Budget line available but challenges exist in sufficient allocation, disbursement and its tracking | Budget line available, allocated sufficiently but challenges in efficient/ adequate disbursement and tracking | Budget line for interventions available, efficiently and sufficiently allocated, efficiently disbursed and processes instituted for tracking, with increasing trend in provisions |
| 1. **Data and information systems** |  |  |  |  |
| Is data collected on these interventions from national surveys and/or national information systems? If “yes” then, does the system include all relevant indicators and is used for program monitoring/quality improvement?   - *Are there specific goals/ targets for coverage assigned at the national level for the intervention?* - *Is data on interventions is collected from national surveys (e.g. DHS or equivalent)?* - *Does the national information system (e.g. Health Management Information System) reporting include relevant indicators pertaining to the specific intervention?* - *Is the data quality maintained through timeliness, periodicity and consistency?* - *Are systems in place for use of data generated from reporting/monitoring for programme review/ quality improvement/ decision-making?* | National Data and Information Systems are not in place/ non-functional | Specific goals/targets for coverage are assigned, however gaps existing in national survey or national information system to include relevant indicators and faces challenges in regular reporting, data quality and data use for decision making | Specific goals/targets for coverage are assigned, data collected through national survey and national information system systems on relevant indicators however some challenges exist around regular reporting, data quality OR data use for decision making | National coverage targets assigned, data collected through national surveys and national information systems with data quality and systems in place for ensuring use of data |
| 1. **Work force** |  |  |  |  |
| Are there defined responsibilities of service providers at all levels and their supervisors for implementing the interventions? Do capacity building plans exist? Adequate numbers?   - *Are there defined SOPs/ responsibilities of service providers and their supervisors at all levels for implementing the interventions?* - *Are at least 50% of staff trained at least once on implementation protocols in last on years?* - *Is the level of vacancies of trained service providers available to deliver interventions less than 25%?* - *Are there functional mechanisms in place for regular assessment and quality improvement of service provider performance?* | Guidelines and operational plans are not well defined/ do not exist at strategic level and operational level, with <25% staff trained, >50% vacancies | Guidelines and operational plans for service providers are available but face challenges in implementation along with two or more of the following: trained staff, level of vacancies, mechanism for performance assessment/ quality improvement | Guidelines and operational plans for service providers are available but face challenges in implementation along with any one of the following: trained staff, level of vacancies, mechanism for performance assessment/ quality improvement | Guidelines for service providers available, >50% staff trained, <25% vacancies of service providers and functional mechanisms for performance assessment/ quality improvement are in place |
| 1. **Essential commodities and supplies** |  |  |  |  |
| Are there national standards, policies in place with respect to procurement and supply of the required drugs and equipment for the interventions? Government-owned, financed and tracking and management of stockouts?   - *Are there national standards and policies in place with respect to the required drugs and equipment?* - *Is in country procurement systems government-owned and -financed for procurement and delivery of commodities and supplies?* - *Are the procedures and mechanisms for storage and distribution of commodities and supplies for related interventions available at levels of health system?* - *Is there a system (Logistics management information system/LMIS) in place to track and manage stockouts of commodities and supplies at all system levels?* | Standard policies for drugs and equipment are not available and systems are non-functional/ need to be effectively implemented | Standard policies for drugs and equipment are in place but challenges are faced in 2 to 3 of the following areas: system for procurement, distribution and storage, systems to manage stockouts | Standard policies for drugs and equipment are in place with appropriate procurement systems but challenges existing in distribution and storage OR systems to manage stockouts | Standard policies for drugs and equipment are in place with a reliable system for procurement, distribution and storage, with equitable access at all levels, systems to manage stockouts |
| 1. **Service delivery** |  |  |  |  |
| Are there frameworks/guidelines for implementation and monitoring the service delivery? Management and referral of cases; appropriate health infrastructure; mechanisms for increasing service demand; effective coverage (equity and compliance)?   - *Are there frameworks/ guidelines for implementation and monitoring of the service delivery for the interventions?* - *Are there process and mechanisms established for management and referral of cases with appropriate service availability at required levels?* - *Is the appropriate health infrastructure in place (physical, utilities supply, ICT and transport/logistics) for better service delivery?* - *Are there mechanisms to influence behaviour change in the community, through rights-based approach and improving accountability of the systems to improve demand for services?* - *Have the services been provided to all population, including the most vulnerable and at-risk groups, in an equitable manner with a defined mechanism and strategy in place?* - *Is there an observed compliance in use of services and interventions delivered to the population?* | Appropriate frameworks/ guidelines for implementation and monitoring of service delivery do not exist and sub-optimal coverage | Frameworks/ guidelines for service delivery, with required health infrastructure, but requires processes for demand for services, equitable access, coverage and compliance at population level | Frameworks/ guidelines for service delivery, with required health infrastructure and processes for demand for services, however challenges exist in equitable access, coverage and compliance at population level | Service delivery with equity and effective coverage exists, supported by required frameworks/ guidelines, health infrastructure, processes of increasing demand of services and observed compliance |

The processes followed for bottleneck analysis include the following:

- Collated of relevant policy and program documents for key evidence-based interventions. These included strategies/plans/policies, national guidelines and standards, reports, reviews, assessments and national survey data.
- Validated the existence/ availability/ alignment of policy and program documents against each recommended intervention in consultation with UNICEF country office representatives.
- Reviewed relevant policy and program documents as well as national survey data to examine the bottlenecks in implementation of each intervention (for those interventions where programs do not exist, systems bottlenecks analysis was not undertaken).
- Conducted consultative meetings with relevant stakeholders from each country to examine the severity of bottlenecks. Stakeholders include representatives from UNICEF country offices, thematic area experts, academicians, and government representatives.
- Computed an average score based on the bottleneck grading given by each respondent for health system component against each intervention.
- Colour coded the relevant component to depict bottleneck severity, based on the average score; lesser the score higher is the severity.

**Supplementary Table S4: Number and types of stakeholders consulted by Country**

| Country | Number of stakeholders | Types of stakeholders |
| --- | --- | --- |
| Afghanistan | 5 | 5 UNICEF personnel |
| Bangladesh | 4 | 1 UNICEF personnel and 3 academic and subject matter experts |
| Bhutan | 3 | 2 UNICEF personnel and 1 Government focal point |
| India | 11 | 2 UNICEF personnel, 2 experts working at national/state Government, and 7 academic and subject matter experts |
| Maldives | 3 | 2 UNICEF personnel, 1 subject matter experts |
| Nepal | 5 | 1 UNICEF personnel, 4 academic and subject matter experts |
| Pakistan | 6 | 4 UNICEF personnel, 2 academic experts |
| Sri Lanka | 6 | 2 UNICEF personnel, 3 experts working at national Government, and 1 academic expert |
| Total | **43** |  |

**S5: Programme availability and severity of system bottlenecks* impeding effective implementation of the preconception nutrition interventions**

***S5a. Afghanistan***

| **Evidence-based nutrition interventions for married non-pregnant women (15-49 years)** | Legislation and policies | **System building blocks** | | | | | | | | |  |
| --- | --- | --- | --- | --- | --- | --- | --- | --- | --- | --- | --- |
|  |  | Leadership, management, coordination and governance | Budget and financing | | Data and information | | Work force | Essential commodities and supplies | | Service delivery |  |
| **I. Health and nutrition screening** |  | | | | | | | | | | |
| - 1. Anthropometry (height, weight, waist-hip ratio) |  |  |  | |  | |  |  | |  |  |
| - 1. Anaemia (Blood test such as haemoglobin) |  |  |  | |  | |  |  | |  |  |
| - 1. Diabetes mellitus using oral glucose tolerance test |  |  |  | |  | |  |  | |  |  |
| - 1. Sexually transmitted infections (STIs) or reproductive tract infections (RTIs) |  |  |  | |  | |  |  | |  |  |
| - 1. Psychosocial problems |  |  |  | |  | |  |  | |  |  |
| **II. Provision of essential micronutrients** |  | | | | | | | | | | |
| - 1. Folic acid supplements, if planning pregnancy (400 μg or 0.4 mg daily) |  |  |  | |  | |  |  | |  |  |
| - 1. Supplementation with iron containing supplements (Iron Folic Acid or IFA) |  |  |  | |  | |  |  | |  |  |
| - 1. Large-scale food fortification of staple foods (e.g., cooking oil, rice, salt and wheat flour) with one or more micronutrients (e.g., folic acid, iron, vitamin A and iodine) |  |  | |  | |  |  | |  |  | |
| **III. Counselling on healthy eating and family planning choices and services** |  | | | | | | | | | | |
| - 1. Counselling on healthy eating and physical activity to attain or maintain a healthy weight |  |  | |  | |  |  | |  |  | |
| - 1. Reduce caffeine/ alcohol/ smoke / toxin exposure |  |  | |  | |  |  | |  |  | |
| - 1. Psychosocial counselling |  |  | |  | |  |  | |  |  | |
| - 1. Family planning choices for delaying age at first pregnancy (>=20 years) / increase inter-birth interval (at least 24 months) |  |  | |  | |  |  | |  |  | |
| **IV. Infection prevention** |  | | | | | | | | | | |
| 1. Deworming prophylaxis |  |  | |  | |  |  | |  |  | |
| 1. Provision of bed nets in malaria endemic areas |  |  | |  | |  |  | |  |  | |
| **V. Treatment and care for women at-risk^1^** |  | | | | | | | | | | |
| 1. Social protection interventions (cash, food voucher, food ration) |  |  | |  | |  |  | |  |  | |
| 1. Balanced energy and protein supplements in women of reproductive age in undernourished populations (underweight >= 20%) |  |  | |  | |  |  | |  |  | |
| 1. Contextualised dietary modification linked counselling tailored to nutrition assessment (under- or overweight/anaemia/diabetes) |  |  | |  | |  |  | |  |  | |
| 1. Medical Nutrition Therapy, based on nutrition risk classification (underweight/ overweight/ anaemia/ diabetes) |  |  | |  | |  |  | |  |  | |
| 1. Anaemia with daily IFA supplementation for a period of 3 months |  |  | |  | |  |  |  | |  |  |
| 1. Diabetes mellitus |  |  | |  | |  |  |  | |  |  |
| 1. Depression and other psychosocial problems |  |  | |  | |  |  |  | |  |  |
| 1. STI/RTI |  |  | |  | |  |  |  | |  |  |

*Classification of the severity of the bottleneck is based on criteria presented in S3.
^1^ Underweight (BMI <18.5 kg/m2); Overweight/obese (BMI >=25 kg/m2); Short stature (height <145 cm); Anaemia (Hb <=12 g/dl); Diabetic (blood sugar level >140 mg/dl ); Having mental health concerns; Having STI/RTIs

| No bottleneck |  |
| --- | --- |
| Mild bottleneck |  |
| Moderate bottleneck |  |
| Significant bottleneck |  |
| No programme |  |
| Programme not needed, as per context |  |

***S5b. Bangladesh***

| **Evidence-based nutrition interventions for married non-pregnant women (15-49 years)** | Legislation and policies | **System building blocks** | | | | | |
| --- | --- | --- | --- | --- | --- | --- | --- |
|  |  | Leadership, management, coordination and governance | Budget and financing | Data and information | Work force | Essential commodities and supplies | Service delivery |
| **I. Health and nutrition screening** |  | | | | | | |
| - 1. Anthropometry (height, weight, waist-hip ratio) |  |  |  |  |  |  |  |
| - 1. Anaemia (Blood test such as haemoglobin) |  |  |  |  |  |  |  |
| - 1. Diabetes mellitus using oral glucose tolerance test |  |  |  |  |  |  |  |
| - 1. Sexually transmitted infections (STIs) or reproductive tract infections (RTIs) |  |  |  |  |  |  |  |
| - 1. Psychosocial problems |  |  |  |  |  |  |  |
| **II. Provision of essential micronutrients** |  | | | | | | |
| - 1. Folic acid supplements, if planning pregnancy (400 μg or 0.4 mg daily) | ​ | ​ | ​ | ​ | ​ | ​ | ​ |
| - 1. Supplementation with iron containing supplements (Iron Folic Acid or IFA) | ​ | ​ | ​ | ​ | ​ | ​ | ​ |
| - 1. Large-scale food fortification of staple foods (e.g., cooking oil, rice, salt and wheat flour) with one or more micronutrients (e.g., folic acid, iron, vitamin A and iodine) | ​ | ​ | ​ | ​ | ​ | ​ | ​ |
| **III. Counselling on healthy eating and family planning choices and services** |  | | | | | | |
| - 1. Counselling on healthy eating and physical activity to attain or maintain a healthy weight | ​ | ​ | ​ | ​ | ​ | ​ | ​ |
| - 1. Reduce caffeine/ alcohol/ smoke / toxin exposure | ​ | ​ | ​ | ​ | ​ | ​ | ​ |
| - 1. Psychosocial counselling |  |  |  |  |  |  |  |
| - 1. Family planning choices for delaying age at first pregnancy (>=20 years) / increase inter-birth interval (at least 24 months) | ​ | ​ | ​ | ​ | ​ | ​ | ​ |
| **IV. Infection prevention** |  | | | | | | |
| 1. Deworming prophylaxis |  |  |  |  |  |  |  |
| 1. Provision of bed nets in malaria endemic areas |  |  |  |  |  |  |  |
| **V. Treatment and care for women at-risk^1^** |  | | | | | | |
| 1. Social protection interventions (cash, food voucher, food ration) |  |  |  |  |  |  |  |
| 1. Balanced energy and protein supplements in women of reproductive age in undernourished populations (underweight >= 20%) |  |  |  |  |  |  |  |
| 1. Contextualised dietary modification linked counselling tailored to nutrition assessment (under- or overweight/anaemia/diabetes) |  |  |  |  |  |  |  |
| 1. Medical Nutrition Therapy, based on nutrition risk classification (underweight/ overweight/ anaemia/ diabetes) |  |  |  |  |  |  |  |
| 1. Anaemia with daily IFA supplementation for a period of 3 months |  |  |  |  |  |  |  |
| 1. Diabetes mellitus |  |  |  |  |  |  |  |
| 1. Depression and other psychosocial problems |  |  |  |  |  |  |  |
| 1. STI/RTI |  |  |  |  |  |  |  |

*Classification of the severity of the bottleneck is based on criteria presented in S3.
^1^ Underweight (BMI <18.5 kg/m2); Overweight/obese (BMI >=25 kg/m2); Short stature (height <145 cm); Anaemia (Hb <=12 g/dl); Diabetic (blood sugar level >140 mg/dl ); Having mental health concerns; Having STI/RTIs

| No bottleneck |  |
| --- | --- |
| Mild bottleneck |  |
| Moderate bottleneck |  |
| Significant bottleneck |  |
| No programme |  |
| Programme not needed, as per context |  |

***S5c. Bhutan***

| **Evidence-based nutrition interventions for married non-pregnant women (15-49 years)** | Legislation and policies | **System building blocks** | | | | | |
| --- | --- | --- | --- | --- | --- | --- | --- |
|  |  | Leadership, management, coordination and governance | Budget and financing | Data and information | Work force | Essential commodities and supplies | Service delivery |
| **I. Health and nutrition screening** |  | | | | | | |
| - 1. Anthropometry (height, weight, waist-hip ratio) |  |  |  |  |  |  |  |
| - 1. Anaemia (Blood test such as haemoglobin) | ​ | ​ | ​ | ​ | ​ | ​ | ​ |
| - 1. Diabetes mellitus using oral glucose tolerance test | ​ | ​ | ​ | ​ | ​ | ​ | ​ |
| - 1. Sexually transmitted infections (STIs) or reproductive tract infections (RTIs) | ​ | ​ | ​ | ​ | ​ | ​ | ​ |
| - 1. Psychosocial problems | ​ | ​ | ​ | ​ | ​ | ​ | ​ |
| **II. Provision of essential micronutrients** |  | | | | | | |
| - 1. Folic acid supplements, if planning pregnancy (400 μg or 0.4 mg daily) | ​ | ​ | ​ | ​ | ​ | ​ | ​ |
| - 1. Supplementation with iron containing supplements (Iron Folic Acid or IFA) | ​ | ​ | ​ | ​ | ​ | ​ | ​ |
| - 1. Large-scale food fortification of staple foods (e.g., cooking oil, rice, salt and wheat flour) with one or more micronutrients (e.g., folic acid, iron, vitamin A and iodine) | ​ | ​ | ​ | ​ | ​ | ​ | ​ |
| **III. Counselling on healthy eating and family planning choices and services** |  | | | | | | |
| - 1. Counselling on healthy eating and physical activity to attain or maintain a healthy weight | ​ | ​ | ​ | ​ | ​ | ​ | ​ |
| - 1. Reduce caffeine/ alcohol/ smoke / toxin exposure | ​ | ​ | ​ | ​ | ​ | ​ | ​ |
| - 1. Psychosocial counselling | ​ | ​ | ​ | ​ | ​ | ​ | ​ |
| - 1. Family planning choices for delaying age at first pregnancy (>=20 years) / increase inter-birth interval (at least 24 months) | ​ | ​ | ​ | ​ | ​ | ​ | ​ |
| **IV. Infection prevention** |  | | | | | | |
| 1. Deworming prophylaxis | ​ | ​ | ​ | ​ | ​ | ​ | ​ |
| 1. Provision of bed nets in malaria endemic areas | ​ | ​ | ​ | ​ | ​ | ​ | ​ |
| **V. Treatment and care for women at-risk^1^** |  | | | | | | |
| 1. Social protection interventions (cash, food voucher, food ration) |  |  |  |  |  |  |  |
| 1. Balanced energy and protein supplements in women of reproductive age in undernourished populations (underweight >= 20%) | ​ |  |  |  |  |  |  |
| 1. Contextualised dietary modification linked counselling tailored to nutrition assessment (under- or overweight/anaemia/diabetes) | ​ | ​ | ​ | ​ | ​ | ​ | ​ |
| 1. Medical Nutrition Therapy, based on nutrition risk classification (underweight/ overweight/ anaemia/ diabetes) | ​ | ​ | ​ | ​ | ​ | ​ | ​ |
| 1. Anaemia with daily IFA supplementation for a period of 3 months | ​ | ​ | ​ | ​ | ​ | ​ | ​ |
| 1. Diabetes mellitus | ​ | ​ | ​ | ​ | ​ | ​ | ​ |
| 1. Depression and other psychosocial problems | ​ | ​ | ​ | ​ | ​ | ​ | ​ |
| 1. STI/RTI | ​ | ​ | ​ | ​ | ​ | ​ | ​ |

*Classification of the severity of the bottleneck is based on criteria presented in S3.
^1^ Underweight (BMI <18.5 kg/m2); Overweight/obese (BMI >=25 kg/m2); Short stature (height <145 cm); Anaemia (Hb <=12 g/dl); Diabetic (blood sugar level >140 mg/dl ); Having mental health concerns; Having STI/RTIs

| No bottleneck |  |
| --- | --- |
| Mild bottleneck |  |
| Moderate bottleneck |  |
| Significant bottleneck |  |
| No programme |  |
| Programme not needed, as per context |  |

***S5d. India***

| **Evidence-based nutrition interventions for married non-pregnant women (15-49 years)** | Legislation and policies | **System building blocks** | | | | | |
| --- | --- | --- | --- | --- | --- | --- | --- |
|  |  | Leadership, management, coordination and governance | Budget and financing | Data and information | Work force | Essential commodities and supplies | Service delivery |
| **I. Health and nutrition screening** |  | | | | | | |
| - 1. Anthropometry (height, weight, waist-hip ratio) | ​ | ​ | ​ | ​ | ​ | ​ | ​ |
| - 1. Anaemia (Blood test such as haemoglobin) | ​ | ​ | ​ | ​ | ​ | ​ | ​ |
| - 1. Diabetes mellitus using oral glucose tolerance test | ​ | ​ | ​ | ​ | ​ | ​ | ​ |
| - 1. Sexually transmitted infections (STIs) or reproductive tract infections (RTIs) | ​ | ​ | ​ | ​ | ​ | ​ | ​ |
| - 1. Psychosocial problems | ​ | ​ | ​ | ​ | ​ | ​ | ​ |
| **II. Provision of essential micronutrients** |  | | | | | | |
| - 1. Folic acid supplements, if planning pregnancy (400 μg or 0.4 mg daily) | ​ | ​ | ​ | ​ | ​ | ​ | ​ |
| - 1. Supplementation with iron containing supplements (Iron Folic Acid or IFA) | ​ | ​ | ​ | ​ | ​ | ​ | ​ |
| - 1. Large-scale food fortification of staple foods (e.g., cooking oil, rice, salt and wheat flour) with one or more micronutrients (e.g., folic acid, iron, vitamin A and iodine) | ​ | ​ | ​ | ​ | ​ | ​ | ​ |
| **III. Counselling on healthy eating and family planning choices and services** |  | | | | | | |
| - 1. Counselling on healthy eating and physical activity to attain or maintain a healthy weight | ​ | ​ | ​ | ​ | ​ | ​ | ​ |
| - 1. Reduce caffeine/ alcohol/ smoke / toxin exposure | ​ | ​ | ​ | ​ | ​ | ​ | ​ |
| - 1. Psychosocial counselling | ​ | ​ | ​ | ​ | ​ | ​ | ​ |
| - 1. Family planning choices for delaying age at first pregnancy (>=20 years) / increase inter-birth interval (at least 24 months) | ​ | ​ | ​ | ​ | ​ | ​ | ​ |
| **IV. Infection prevention** |  | | | | | | |
| 1. Deworming prophylaxis | ​ | ​ | ​ | ​ | ​ | ​ | ​ |
| 1. Provision of bed nets in malaria endemic areas | ​ | ​ | ​ | ​ | ​ | ​ | ​ |
| **V. Treatment and care for women at-risk^1^** |  | | | | | | |
| 1. Social protection interventions (cash, food voucher, food ration) |  |  |  |  |  |  |  |
| 1. Balanced energy and protein supplements in women of reproductive age in undernourished populations (underweight >= 20%) | ​ |  |  |  |  |  |  |
| 1. Contextualised dietary modification linked counselling tailored to nutrition assessment (under- or overweight/anaemia/diabetes) | ​ | ​ | ​ | ​ | ​ | ​ | ​ |
| 1. Medical Nutrition Therapy, based on nutrition risk classification (underweight/ overweight/ anaemia/ diabetes) |  |  |  |  |  |  |  |
| 1. Anaemia with daily IFA supplementation for a period of 3 months | ​ | ​ | ​ | ​ | ​ | ​ | ​ |
| 1. Diabetes mellitus | ​ | ​ | ​ | ​ | ​ | ​ | ​ |
| 1. Depression and other psychosocial problems | ​ | ​ | ​ | ​ | ​ | ​ | ​ |
| 1. STI/RTI | ​ | ​ | ​ | ​ | ​ | ​ | ​ |

*Classification of the severity of the bottleneck is based on criteria presented in S3.
^1^ Underweight (BMI <18.5 kg/m2); Overweight/obese (BMI >=25 kg/m2); Short stature (height <145 cm); Anaemia (Hb <=12 g/dl); Diabetic (blood sugar level >140 mg/dl ); Having mental health concerns; Having STI/RTIs

| No bottleneck |  |
| --- | --- |
| Mild bottleneck |  |
| Moderate bottleneck |  |
| Significant bottleneck |  |
| No programme |  |
| Programme not needed, as per context |  |

***S5e. Maldives***

| **Evidence-based nutrition interventions for married non-pregnant women (15-49 years)** | Legislation and policies | **System building blocks** | | | | | |
| --- | --- | --- | --- | --- | --- | --- | --- |
|  |  | Leadership, management, coordination and governance | Budget and financing | Data and information | Work force | Essential commodities and supplies | Service delivery |
| **I. Health and nutrition screening** |  | | | | | | |
| - 1. Anthropometry (height, weight, waist-hip ratio) | ​ | ​ | ​ | ​ | ​ | ​ | ​ |
| - 1. Anaemia (Blood test such as haemoglobin) | ​ |  |  |  |  |  |  |
| - 1. Diabetes mellitus using oral glucose tolerance test | ​ | ​ | ​ | ​ | ​ | ​ | ​ |
| - 1. Sexually transmitted infections (STIs) or reproductive tract infections (RTIs) | ​ | ​ | ​ | ​ | ​ | ​ | ​ |
| - 1. Psychosocial problems | ​ | ​ | ​ | ​ | ​ | ​ | ​ |
| **II. Provision of essential micronutrients** |  | | | | | | |
| - 1. Folic acid supplements, if planning pregnancy (400 μg or 0.4 mg daily) |  |  |  |  |  |  |  |
| - 1. Supplementation with iron containing supplements (Iron Folic Acid or IFA) | ​ | ​ | ​ | ​ | ​ | ​ | ​ |
| - 1. Large-scale food fortification of staple foods (e.g., cooking oil, rice, salt and wheat flour) with one or more micronutrients (e.g., folic acid, iron, vitamin A and iodine) | ​ | ​ | ​ | ​ | ​ | ​ | ​ |
| **III. Counselling on healthy eating and family planning choices and services** |  | | | | | | |
| - 1. Counselling on healthy eating and physical activity to attain or maintain a healthy weight | ​ | ​ | ​ | ​ | ​ | ​ | ​ |
| - 1. Reduce caffeine/ alcohol/ smoke/ toxin exposure | ​ | ​ | ​ | ​ | ​ | ​ | ​ |
| - 1. Psychosocial counselling | ​ | ​ | ​ | ​ | ​ | ​ | ​ |
| - 1. Family planning choices for delaying age at first pregnancy (>=20 years) / increase inter-birth interval (at least 24 months) | ​ | ​ | ​ | ​ | ​ | ​ | ​ |
| **IV. Infection prevention** |  | | | | | | |
| 1. Deworming prophylaxis |  |  |  |  |  |  |  |
| 1. Provision of bed nets in malaria endemic areas |  | ​ | ​ | ​ | ​ | ​ | ​ |
| **V. Treatment and care for women at-risk^1^** |  |  |  |  |  |  |  |
| 1. Social protection interventions (cash, food voucher, food ration) |  |  |  |  |  |  |  |
| 1. Balanced energy and protein supplements in women of reproductive age in undernourished populations (underweight >= 20%) |  |  |  |  |  |  |  |
| 1. Contextualised dietary modification linked counselling tailored to nutrition assessment (under- or overweight/anaemia/diabetes) | ​ | ​ | ​ | ​ | ​ | ​ | ​ |
| 1. Medical Nutrition Therapy, based on nutrition risk classification (underweight/ overweight/ anaemia/ diabetes) |  |  |  |  |  |  |  |
| 1. Anaemia with daily IFA supplementation for a period of 3 months |  |  |  |  |  |  |  |
| 1. Diabetes mellitus | ​ | ​ | ​ | ​ | ​ | ​ | ​ |
| 1. Depression and other psychosocial problems | ​ | ​ | ​ | ​ | ​ | ​ | ​ |
| 1. STI/RTI | ​ | ​ | ​ | ​ | ​ | ​ | ​ |

*Classification of the severity of the bottleneck is based on criteria presented in S3.
^1^ Underweight (BMI <18.5 kg/m2); Overweight/obese (BMI >=25 kg/m2); Short stature (height <145 cm); Anaemia (Hb <=12 g/dl); Diabetic (blood sugar level >140 mg/dl ); Having mental health concerns; Having STI/RTIs

| No bottleneck |  |
| --- | --- |
| Mild bottleneck |  |
| Moderate bottleneck |  |
| Significant bottleneck |  |
| No programme |  |
| Programme not needed, as per context |  |

***S5f. Nepal***

| **Evidence-based nutrition interventions for married non-pregnant women (15-49 years)** | Legislation and policies | **System building blocks** | | | | | |
| --- | --- | --- | --- | --- | --- | --- | --- |
|  |  | Leadership, management, coordination and governance | Budget and financing | Data and information | Work force | Essential commodities and supplies | Service delivery |
| **I. Health and nutrition screening** |  | | | | | | |
| - 1. Anthropometry (height, weight, waist-hip ratio) | ​ | ​ | ​ | ​ | ​ | ​ | ​ |
| - 1. Anaemia (Blood test such as haemoglobin) | ​ | ​ | ​ | ​ | ​ | ​ | ​ |
| - 1. Diabetes mellitus using oral glucose tolerance test | ​ | ​ | ​ | ​ | ​ | ​ | ​ |
| - 1. Sexually transmitted infections (STIs) or reproductive tract infections (RTIs) | ​ | ​ | ​ | ​ | ​ | ​ | ​ |
| - 1. Psychosocial problems | ​ | ​ | ​ | ​ | ​ | ​ | ​ |
| **II. Provision of essential micronutrients** |  | | | | | | |
| - 1. Folic acid supplements, if planning pregnancy (400 μg or 0.4 mg daily) | ​ | ​ | ​ | ​ | ​ | ​ | ​ |
| - 1. Supplementation with iron containing supplements (Iron Folic Acid or IFA) | ​ | ​ | ​ | ​ | ​ | ​ | ​ |
| - 1. Large-scale food fortification of staple foods (e.g., cooking oil, rice, salt and wheat flour) with one or more micronutrients (e.g., folic acid, iron, vitamin A and iodine) | ​ | ​ | ​ | ​ | ​ | ​ | ​ |
| **III. Counselling on healthy eating and family planning choices and services** |  | | | | | | |
| - 1. Counselling on healthy eating and physical activity to attain or maintain a healthy weight | ​ | ​ | ​ | ​ | ​ | ​ | ​ |
| - 1. Reduce caffeine/ alcohol/ smoke / toxin exposure | ​ | ​ | ​ | ​ | ​ | ​ | ​ |
| - 1. Psychosocial counselling | ​ | ​ | ​ | ​ | ​ | ​ | ​ |
| - 1. Family planning choices for delaying age at first pregnancy (>=20 years) / increase inter-birth interval (at least 24 months) | ​ | ​ | ​ | ​ | ​ | ​ | ​ |
| **IV. Infection prevention** |  | | | | | | |
| 1. Deworming prophylaxis | ​ | ​ | ​ | ​ | ​ | ​ | ​ |
| 1. Provision of bed nets in malaria endemic areas | ​ | ​ | ​ | ​ | ​ | ​ | ​ |
| **V. Treatment and care for women at-risk^1^** |  | | | | | | |
| 1. Social protection interventions (cash, food voucher, food ration) |  |  |  |  |  |  |  |
| 1. Balanced energy and protein supplements in women of reproductive age in undernourished populations (underweight >= 20%) | ​ |  |  |  |  |  |  |
| 1. Contextualised dietary modification linked counselling tailored to nutrition assessment (under- or overweight/anaemia/diabetes) |  |  |  |  |  |  |  |
| 1. Medical Nutrition Therapy, based on nutrition risk classification (underweight/ overweight/ anaemia/ diabetes) |  |  |  |  |  |  |  |
| 1. Anaemia with daily IFA supplementation for a period of 3 months |  |  |  |  |  |  |  |
| 1. Diabetes mellitus | ​ | ​ | ​ | ​ | ​ | ​ | ​ |
| 1. Depression and other psychosocial problems | ​ | ​ | ​ | ​ | ​ | ​ | ​ |
| 1. STI/RTI | ​ | ​ | ​ | ​ | ​ | ​ | ​ |

*Classification of the severity of the bottleneck is based on criteria presented in S3.
^1^ Underweight (BMI <18.5 kg/m2); Overweight/obese (BMI >=25 kg/m2); Short stature (height <145 cm); Anaemia (Hb <=12 g/dl); Diabetic (blood sugar level >140 mg/dl ); Having mental health concerns; Having STI/RTIs

| No bottleneck |  |
| --- | --- |
| Mild bottleneck |  |
| Moderate bottleneck |  |
| Significant bottleneck |  |
| No programme |  |
| Programme not needed, as per context |  |

***S5g. Pakistan***

| **Evidence-based nutrition interventions for married non-pregnant women (15-49 years)** | Legislation and policies | **System building blocks** | | | | | |
| --- | --- | --- | --- | --- | --- | --- | --- |
|  |  | Leadership, management, coordination and governance | Budget and financing | Data and information | Work force | Essential commodities and supplies | Service delivery |
| **I. Health and nutrition screening** |  | | | | | | |
| - 1. Anthropometry (height, weight, waist-hip ratio) | ​ |  |  |  |  |  |  |
| - 1. Anaemia (Blood test such as haemoglobin) | ​ |  |  |  |  |  |  |
| - 1. Diabetes mellitus using oral glucose tolerance test | ​ |  |  |  |  |  |  |
| - 1. Sexually transmitted infections (STIs) or reproductive tract infections (RTIs) | ​ | ​ | ​ | ​ | ​ | ​ | ​ |
| - 1. Psychosocial problems | ​ |  |  |  |  |  |  |
| **II. Provision of essential micronutrients** |  | | | | | | |
| - 1. Folic acid supplements, if planning pregnancy (400 μg or 0.4 mg daily) | ​ |  |  |  |  |  |  |
| - 1. Supplementation with iron containing supplements (Iron Folic Acid or IFA) | ​ |  |  |  |  |  |  |
| - 1. Large-scale food fortification of staple foods (e.g., cooking oil, rice, salt and wheat flour) with one or more micronutrients (e.g., folic acid, iron, vitamin A and iodine) | ​ | ​ | ​ | ​ | ​ | ​ | ​ |
| **III. Counselling on healthy eating and family planning choices and services** |  | | | | | | |
| - 1. Counselling on healthy eating and physical activity to attain or maintain a healthy weight | ​ |  |  |  |  |  |  |
| - 1. Reduce caffeine/ alcohol/ smoke / toxin exposure |  |  |  |  |  |  |  |
| - 1. Psychosocial counselling |  |  |  |  |  |  |  |
| - 1. Family planning choices for delaying age at first pregnancy (>=20 years) / increase inter-birth interval (at least 24 months) | ​ | ​ | ​ | ​ | ​ |  | ​ |
| **IV. Infection prevention** | ​ | | | | | | |
| 1. Deworming prophylaxis | ​ |  |  |  |  |  |  |
| 1. Provision of bed nets in malaria endemic areas | ​ | ​ | ​ | ​ | ​ | ​ | ​ |
| **V. Treatment and care for women at-risk^1^** |  | | | | | | |
| 1. Social protection interventions (cash, food voucher, food ration) |  |  |  |  |  |  |  |
| 1. Balanced energy and protein supplements in women of reproductive age in undernourished populations (underweight >= 20%) | ​ |  |  |  |  |  |  |
| 1. Contextualised dietary modification linked counselling tailored to nutrition assessment (under- or overweight/anaemia/diabetes) | ​ |  |  |  |  |  |  |
| 1. Medical Nutrition Therapy, based on nutrition risk classification (underweight/ overweight/ anaemia/ diabetes) |  |  |  |  |  |  |  |
| 1. Anaemia with daily IFA supplementation for a period of 3 months | ​ |  |  |  |  |  |  |
| 1. Diabetes mellitus | ​ |  |  |  |  |  |  |
| 1. Depression and other psychosocial problems | ​ | ​ | ​ | ​ | ​ | ​ | ​ |
| 1. STI/RTI | ​ | ​ | ​ | ​ | ​ | ​ | ​ |

*Classification of the severity of the bottleneck is based on criteria presented in S3.
^1^ Underweight (BMI <18.5 kg/m2); Overweight/obese (BMI >=25 kg/m2); Short stature (height <145 cm); Anaemia (Hb <=12 g/dl); Diabetic (blood sugar level >140 mg/dl ); Having mental health concerns; Having STI/RTIs

| No bottleneck |  |
| --- | --- |
| Mild bottleneck |  |
| Moderate bottleneck |  |
| Significant bottleneck |  |
| No programme |  |
| Programme not needed, as per context |  |

***S5h. Sri Lanka***

| **Evidence-based nutrition interventions for married non-pregnant women (15-49 years)** | Legislation and policies | **System building blocks** | | | | | |
| --- | --- | --- | --- | --- | --- | --- | --- |
|  |  | Leadership, management, coordination and governance | Budget and financing | Data and information | Work force | Essential commodities and supplies | Service delivery |
| **I. Health and nutrition screening** |  | | | | | | |
| - 1. Anthropometry (height, weight, waist-hip ratio) | ​ | ​ | ​ | ​ | ​ | ​ | ​ |
| - 1. Anaemia (Blood test such as haemoglobin) | ​ | ​ | ​ | ​ | ​ | ​ | ​ |
| - 1. Diabetes mellitus using oral glucose tolerance test | ​ | ​ | ​ | ​ | ​ | ​ | ​ |
| - 1. Sexually transmitted infections (STIs) or reproductive tract infections (RTIs) | ​ | ​ | ​ | ​ | ​ | ​ | ​ |
| - 1. Psychosocial problems | ​ | ​ | ​ | ​ | ​ | ​ | ​ |
| **II. Provision of essential micronutrients** |  | | | | | | |
| - 1. Folic acid supplements, if planning pregnancy (400 μg or 0.4 mg daily) | ​ | ​ | ​ | ​ | ​ | ​ | ​ |
| - 1. Supplementation with iron containing supplements (Iron Folic Acid or IFA) |  |  |  |  |  |  |  |
| - 1. Large-scale food fortification of staple foods (e.g., cooking oil, rice, salt and wheat flour) with one or more micronutrients (e.g., folic acid, iron, vitamin A and iodine) | ​ | ​ | ​ | ​ | ​ | ​ | ​ |
| **III. Counselling on healthy eating and family planning choices and services** |  | | | | | | |
| - 1. Counselling on healthy eating and physical activity to attain or maintain a healthy weight | ​ | ​ | ​ | ​ | ​ | ​ | ​ |
| - 1. Reduce caffeine/ alcohol/ smoke / toxin exposure | ​ | ​ | ​ | ​ | ​ | ​ | ​ |
| - 1. Psychosocial counselling | ​ | ​ | ​ | ​ | ​ | ​ | ​ |
| - 1. Family planning choices for delaying age at first pregnancy (>=20 years) / increase inter-birth interval (at least 24 months) | ​ | ​ | ​ | ​ | ​ | ​ | ​ |
| **IV. Infection prevention** |  | | | | | | |
| 1. Deworming prophylaxis |  | ​ | ​ | ​ | ​ | ​ | ​ |
| 1. Provision of bed nets in malaria endemic areas |  | ​ | ​ | ​ | ​ | ​ | ​ |
| **V. Treatment and care for women at-risk^1^** |  | | | | | | |
| 1. Social protection interventions (cash, food voucher, food ration) |  |  |  |  |  |  |  |
| 1. Balanced energy and protein supplements in women of reproductive age in undernourished populations (underweight >= 20%) |  |  |  |  |  |  |  |
| 1. Contextualised dietary modification linked counselling tailored to nutrition assessment (under- or overweight/anaemia/diabetes) | ​ | ​ | ​ | ​ | ​ | ​ | ​ |
| 1. Medical Nutrition Therapy, based on nutrition risk classification (underweight/ overweight/ anaemia/ diabetes) | ​ | ​ | ​ | ​ | ​ | ​ | ​ |
| 1. Anaemia with daily IFA supplementation for a period of 3 months | ​ | ​ | ​ | ​ | ​ | ​ | ​ |
| 1. Diabetes mellitus | ​ | ​ | ​ | ​ | ​ | ​ | ​ |
| 1. Depression and other psychosocial problems | ​ | ​ | ​ | ​ | ​ | ​ | ​ |
| 1. STI/RTI | ​ | ​ | ​ | ​ | ​ | ​ | ​ |

*Classification of the severity of the bottleneck is based on criteria presented in S3.
^1^ Underweight (BMI <18.5 kg/m2); Overweight/obese (BMI >=25 kg/m2); Short stature (height <145 cm); Anaemia (Hb <=12 g/dl); Diabetic (blood sugar level >140 mg/dl ); Having mental health concerns; Having STI/RTIs

| No bottleneck |  |
| --- | --- |
| Mild bottleneck |  |
| Moderate bottleneck |  |
| Significant bottleneck |  |
| No programme |  |
| Programme not needed, as per context |  |
